# Design, Optimization and Mechanistic Insights Into Terpenoid‐Derived Inhibitors of Protein Kinase C Iota

**DOI:** 10.1111/cbdd.70348

**Published:** 2026-06-26

**Authors:** Rachid Chahboun, Fernando Rodríguez‐Serrano, Ángel Bueno, Nuria Mut‐Salud, Ramón Alvarez‐Manzaneda, Enrique Alvarez‐Manzaneda, Antonio Fernández

**Affiliations:** ^1^ Department of Organic Chemistry, Faculty of Sciences, Institute of Biotechnology University of Granada Granada Spain; ^2^ Biopathology and Regenerative Medicine Institute (IBIMER) University of Granada Granada Spain; ^3^ Instituto de Investigación Biosanitaria de Granada (ibs.GRANADA) Granada Spain; ^4^ Area of Organic Chemistry, Department of Chemistry and Physics University of Almería Almería Spain

**Keywords:** cancer drug discovery, kinase inhibition, molecular docking, protein kinase C iota (PKCι), structure–activity relationship (SAR)

## Abstract

Protein kinase C iota (PKCι) is an atypical PKC isoform overexpressed in several human cancers and associated with tumor initiation, maintenance, and resistance to therapy. Meroxest, a synthetic naphthoquinone merosesquiterpene previously shown to exert antitumor activity in breast cancer models, has an incompletely defined mechanism of action. Here, we profiled meroxest against a 410‐kinase panel and synthesized a focused set of analogues to obtain preliminary PKCι‐oriented structure–activity information. At 10 μM, meroxest reduced PKCι enzymatic activity to 52% of control and Cdc7/cyclin B1 activity to 63%, identifying PKCι as the most inhibited kinase in the panel while also indicating that additional kinase targets may contribute to the biological activity of this scaffold. Docking studies supported a plausible interaction of meroxest within the PKCι catalytic site, including hydrogen‐bond contacts with Gly398 and Phe423 consistent with an ATP‐competitive binding mode. Biochemical evaluation of a focused library against PKCι at 10 μM showed that preservation of the quinone framework was important for activity, whereas most structural modifications produced only limited changes under the assay conditions used. Compound **19**, lacking the methyl substituent on the naphthoquinone ring, showed a modest improvement relative to meroxest. Overall, these results support PKCι as a leading candidate target emerging from the meroxest kinase profile and identify compound **19** as a scaffold for further optimization. Future studies will focus on further optimization to improve the modest PKCι inhibitory potency, evaluate activity against Cdc7/cyclin B1 beyond the parent compound, and validate target engagement in cell‐based assays, aiming to identify optimal candidates.

## Graphical abstract

In this study, we identified a synthetic naphthoquinone merosesquiterpene as a potent PKCι inhibitor, emerging as the most strongly inhibited kinase within a panel of 410 human kinases. Structure–activity relationship (SAR) studies using a focused library of terpenoid analogues highlighted the critical role of the naphthoquinone core and the aromatic ring in PKCι inhibition, and revealed an optimized, promising lead scaffold for further development as a PKCι inhibitor.

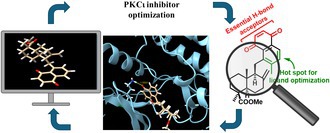

## 1 Introduction

Cancer remains one of the leading causes of morbidity and mortality worldwide and is particularly difficult to treat because tumor cells frequently develop resistance to conventional therapies. As a consequence, recent drug discovery efforts have focused on small‐molecule agents that selectively target dysregulated signaling pathways essential for tumor initiation and progression (Hossain 2024). Within this context, the protein kinase C (PKC) family has emerged as an important group of serine/threonine kinases involved in the regulation of cell proliferation, differentiation, survival, and migration, and aberrant PKC signaling has been implicated in oncogenesis and therapy resistance in several human malignancies (Li et al. 2025). Among these isoforms, atypical protein kinase C iota (PKCι) is frequently overexpressed in diverse tumor types, supporting transformed growth and resistance to therapy (Fields et al. 2007; Regala et al. 2008; Wang et al. 2013). In pancreatic ductal adenocarcinoma (PDAC), PKCι drives transformed growth through RAC‐MEK–ERK signaling and contributes to tumorigenesis and adverse clinical outcome (Scotti et al. 2010; Inman et al. 2022). These observations support PKCι as an attractive molecular target for the development of selective small‐molecule inhibitors (Li et al. 2025).

**FIGURE 1:**
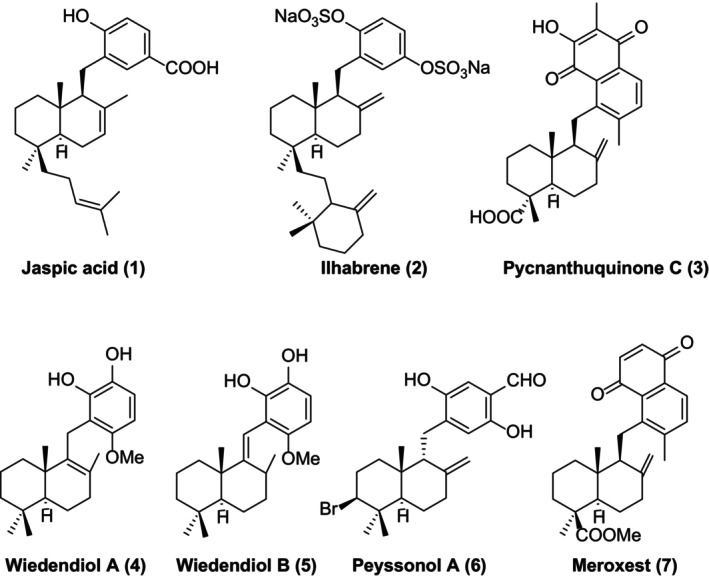
Representative natural naphthoquinones with potent biological activities.

Natural products provide a valuable source of bioactive compounds and molecular scaffolds for anticancer drug discovery. Terpenoid derivatives containing quinone or phenolic moieties, often referred to as meroterpenoids or merosesquiterpenes, exhibit considerable structural diversity and a wide range of biological activities (Tian et al. 2023). Representative examples include jaspic acid (**1**), a human 15‐lipoxygenase (15‐HLO) inhibitor originally isolated from the marine sponge *Jaspis cf. johnstoni* (Murray et al. 1997; Carroll et al. 2001); the disulfated meroterpenoid ilhabrene (**2**), isolated from the Brazilian marine sponge *Callyspongia* sp., which inhibits *Leishmania* spp. adenosine phosphoribosyl transferase (APRT), an antileishmaniasis target (Gray et al. 2006); the fungitoxic terpenoid‐quinone pycnanthuquinone C (**3**) (Wabo et al. 2007); and the drimenyl phenols wiedendiol A (**4**) and wiedendiol B (**5**), identified as potent cholesteryl ester transfer protein (CETP) inhibitors (Coval et al. 1995). In addition, the brominated sesquiterpene peyssonol A (**6**), isolated from the red marine alga *Peyssonnelia* sp., has been described as a potent inhibitor of HIV‐1 reverse transcriptase (Talpir et al. 1994) (Figure 1). These examples illustrate the potential of terpenoid‐based quinone and quinol frameworks as useful starting points for the development of selective enzyme inhibitors with therapeutic relevance.

Inspired by these bioactive merosesquiterpene scaffolds, we previously synthesized a series of natural and synthetic merosesquiterpenes and evaluated their antitumor properties (Carrasco et al. 2014). This work led to the discovery of a synthetic naphthoquinone merosesquiterpene, designated meroxest (**7**; compound **13** in the original series), which showed potent cytotoxic activity against breast cancer cells, as well as activity against colon and lung cancer cell lines. Mechanistic studies demonstrated that meroxest induces oxidative stress, causes G_0_‐G_1_ cell‐cycle arrest associated with cyclin D1 downregulation and pRB hypophosphorylation, increases p27 and p53 expression, and triggers apoptosis accompanied by PARP cleavage. Meroxest (**7**) also modulates epithelial‐mesenchymal transition markers, with increased E‐cadherin and β‐catenin expression. In an immunocompetent C57BL/6 mouse model bearing E0771 breast cancer allografts, meroxest significantly reduced tumor volume and decreased leukocyte infiltration, as well as the expression of Ki67 and VEGF, thereby improving histopathological and molecular prognostic parameters (Carrasco et al. 2014) (Carrasco et al. 2016). Despite this favorable preclinical profile, the direct molecular target of meroxest (**7**) has not yet been identified, and therefore no structure–activity optimization has been carried out in relation to that target.

In the present study, we aimed to gain mechanistic insight into the mode of action of meroxest (**7**) and to guide early scaffold optimization. First, we performed a broad kinase profiling assay, which identified PKCι as the most strongly inhibited kinase within a panel of 410 human kinases, while also revealing relevant inhibition of Cdc7/cyclin B1. Guided by the prominence of PKCι in this screen, and taking advantage of the available crystal structure of PKCι, we carried out molecular docking analyses to characterize a plausible binding mode of meroxest and to define key interactions (Messerschmidt et al. 2005; Hanks and Hunter 1995; Huse and Kuriyan 2002; Knight and Shokat 2005; Manning et al. 2002; Zhang et al. 2009). We then designed and synthesized a focused library of meroxest‐derived terpenoid naphthoquinones bearing systematic modifications in both the quinone core and the peripheral aromatic ring and evaluated their inhibitory activity against PKCι to obtain preliminary SAR information. The aims of this study were therefore (i) to identify candidate kinase targets of meroxest within a broad kinase panel; (ii) to provide preliminary mechanistic evidence supporting PKCι as a leading candidate target emerging from this screen; and (iii) to design, synthesize and characterize a series of meroxest‐derived terpenoid analogues for exploratory PKCι‐directed optimization. This study was designed as a biochemical and structure‐based follow‐up of the kinase profiling results and was not intended to provide a full cellular validation of target engagement, which will require additional work in cancer cell models.

## 2 Materials and Methods

### 2.1 Synthesis of Compounds

Commercial reagents and solvents were purchased from chemical suppliers and used without further purification. Reactions were monitored via thin‐layer chromatography silica plates (E. Merck silica gel 60 F254 precoated plates) (0.25 mm) with ultraviolet (UV) detection (254 or 365 nm) and phosphomolybdic acid solution staining with heating for compound visualization. Specific rotation measurements were carried out on a PerkinElmer 341 polarimeter using a 1 dm length cell and CHCl_3_ as solvent. Infrared (IR) spectra were recorded as thin films or solids on a PerkinElmer model One FTIR spectrophotometer, with samples between NaCl plates or as KBr pellets, and are reported as absorption frequencies (cm^−1^). ^1^H and ^13^C NMR spectra were recorded on BRUKER Nanobay Avance III HD instruments (at 400 or 500 MHz and 100 or 125 MHz, respectively). CDCl_3_ was treated with K_2_CO_3_. Chemical shifts are expressed in parts per million (ppm) and are referenced to CHCl_3_ (7.26 ppm) in ^1^H NMR spectra and CDCl_3_ (77.16 ppm) in ^13^C NMR spectra. High‐resolution mass spectra (HRMS) were recorded on a Waters Xevo G2‐XS QTof spectrometer using Q‐TOF analyzer and ESI^+^ ionization. Flash chromatography was performed on silica gel (Merck Kieselgel 60, 230–400 mesh). Chromatographic separations were carried out by conventional column chromatography on silica gel 60 (230–400 mesh) using EtOAc/hexane mixtures of increasing polarity. Unless otherwise stated, reactions were performed in oven‐dried glassware under an argon atmosphere using dry solvents. Synthetic procedures and characterization data for compounds **9**, **10**, and **14**–**19** are available in the Supporting Information. All compounds tested in the biochemical assays showed a purity ≥ 95%, determined by HPLC.

### 2.2 Kinase Selectivity Determination

Profiling of a 410‐member human kinase panel was performed by Eurofins Discovery using the KinaseProfiler platform (Davis et al. 2011). Eurofins kinase enzymatic assays were carried out at the ATP Km, in duplicate, for a panel of 410 kinases at a single concentration of 10 μM for Meroxest (**7**). Details about the substrate used, protein constructs, controls, and assay protocol for each kinase assay can be found at https://www.eurofinsdiscoveryservices.com.

### 2.3 PKCι Activity Inhibition Study

PKCι inhibitory activity was evaluated using the Eurofins KinaseProfiler/LeadHunter profiling technology. Eurofins radiometric kinase enzymatic assays were carried out at the Km of ATP in duplicate at a test compound concentration of 10 μM for compounds **7, 9, 10, 14, 15, 17**, and **19**. Details about the substrate used, protein constructs, controls, and assay protocol for each kinase assay can be found at https://www.eurofinsdiscoveryservices.com.

### 2.4 Molecular Docking Simulations

Molecular docking studies were performed using SwissDock (https://www.swissdock.ch/), which implements the Attracting Cavities approach for protein‐ligand docking (Grosdidier et al. 2011; Zoete et al. 2016; Röhrig et al. 2023), to explore the binding mode of the designed terpenoid derivatives toward the catalytic domain of PKCι. Ligands were provided as SMILES input, and the crystal structure of PKCι obtained from the Protein Data Bank (PDB ID: 1ZRZ) was used as the receptor (Messerschmidt et al. 2005). Docking was conducted employing the Attracting Cavities 2.0 option to restrict sampling to energetically favorable pockets within the kinase domain. This approach allowed a focused exploration of plausible ligand orientations inside the ATP‐binding cleft and adjacent regulatory subpockets. The resulting clusters were ranked according to the SwissParam Score function, and the best‐scoring poses were selected for further analysis. Key molecular contacts, including hydrogen bonding, hydrophobic interactions, and aromatic stacking, were examined using UCSF ChimeraX to identify residues potentially involved in ligand stabilization within the PKCι catalytic site.

## 3 Results and Discussion

### 3.1 Kinase Activity Inhibition Screening

**FIGURE 2:**
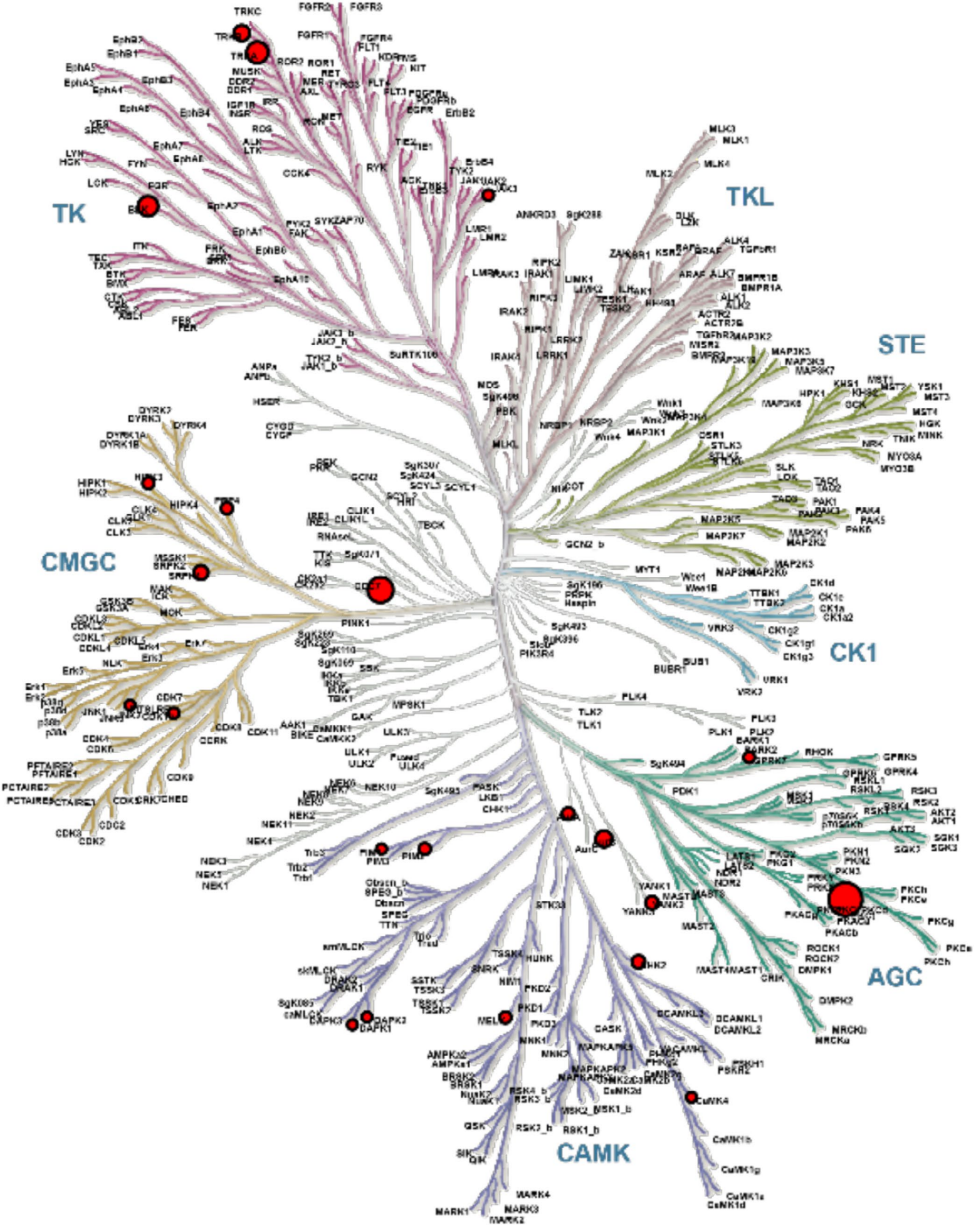
Kinase treespot activity map generated using Morpheus (Broad Institute 2025). The map summarizes the kinase profiling results obtained for meroxest (**7**) at 10 μM. The largest red dot corresponds to PKCι, which showed 52% residual enzymatic activity relative to control.

To gain insight into the antitumor mechanism of meroxest (**7**), we initially performed a comprehensive kinase profiling assay covering 410 protein kinases using the Eurofins KinaseProfiler platform (Figure 2, Table S1). At 10 μM, meroxest (**7**) exhibited minimal effect on most kinases in the panel but reduced the activity of a limited number of enzymes. Among them, PKCι was the most strongly affected kinase, with residual enzymatic activity reduced to 52% of control levels. The screening data also revealed relevant inhibition of Cdc7/cyclin B1, which retained 63% of control activity. Thus, although PKCι emerged as the leading kinase hit and was prioritized for follow‐up biochemical and docking studies, the profiling results suggest a broader mechanism of action rather than an exclusive single‐target mechanism. In this context, meroxest (**7**) may engage multiple kinase targets, with inhibition of Cdc7/cyclin B1 potentially contributing to the biological activity of this scaffold.

PKCι was selected for further study because it was the top hit in the kinase panel and because structural information was available to support docking‐guided analysis. PKCι is a well‐established oncogenic kinase implicated in transformed growth, survival signaling, and therapy resistance in several malignancies, including pancreatic ductal adenocarcinoma (Scotti et al. 2010; Inman et al. 2022; Wang et al. 2013). Beyond pancreatic cancer, PKCι has also been implicated in breast cancer. Functional studies have shown that the selective PKCι inhibitor ICA‐1S reduces proliferation and induces apoptosis in both triple‐negative (BT‐549) and luminal A, estrogen/progesterone receptor‐positive (MCF‐7) breast cancer cells. In addition, pharmacologic or siRNA‐mediated inhibition of PKCι reduced the expression of MAPK/JNK pathway proteins, c‐Jun, and TNF‐α, supporting a role for PKCι in mitogenic and survival signaling across breast cancer subtypes, including hormone receptor‐positive disease (Nowshin Oishee et al. 2025).

Taken together, these findings support PKCι as a leading candidate target emerging from the meroxest (**7**) kinase profile. However, because Cdc7/cyclin B1 also showed relevant inhibition and was not included in the follow‐up analysis of the analogue series, the present data should be interpreted as defining a PKCι‐prioritized mechanistic direction rather than a complete deconvolution of the kinase selectivity profile of this scaffold.

### 3.2 Molecular Docking Studies of Meroxest

**FIGURE 3:**
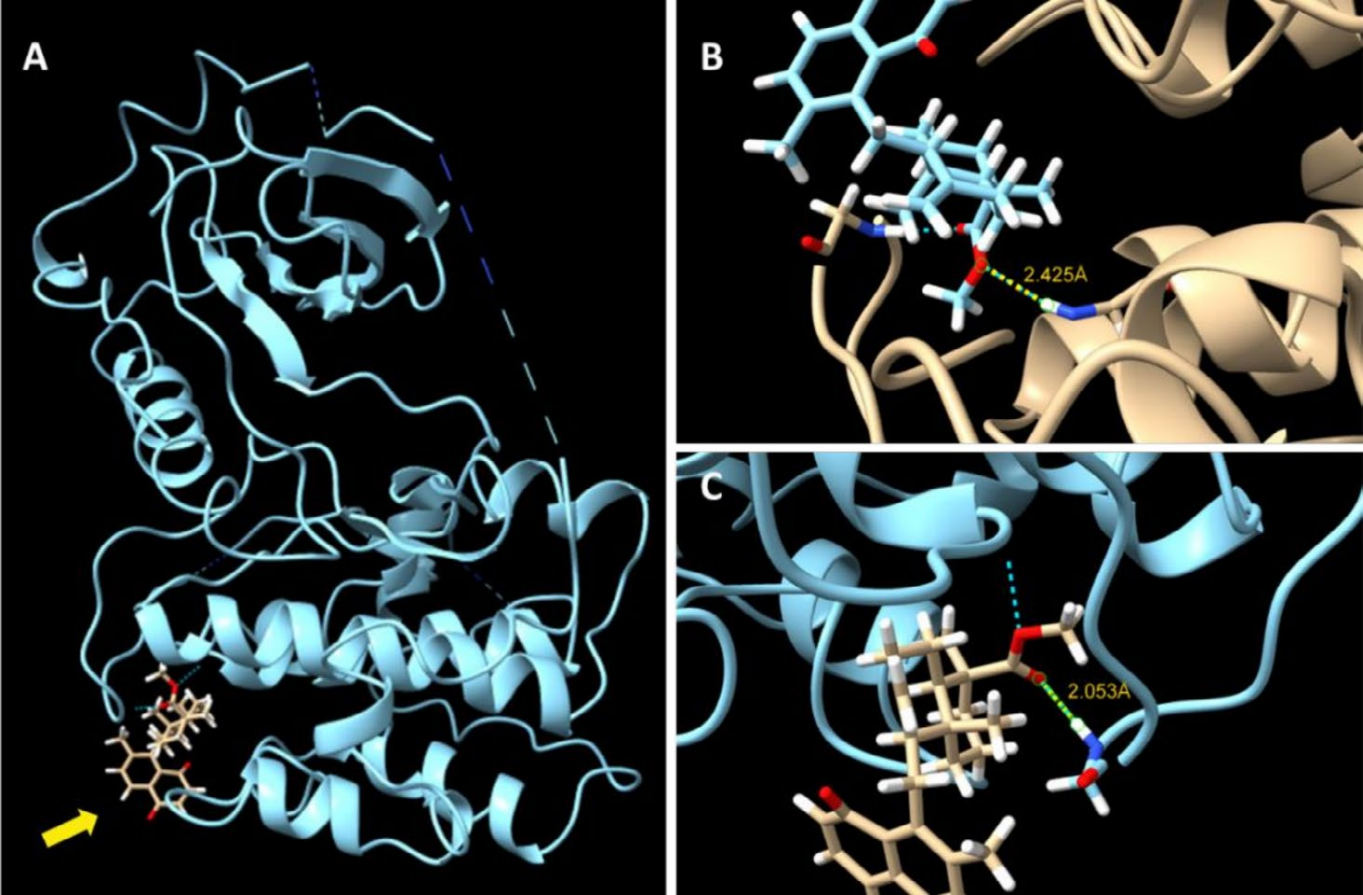
Graphical results of the molecular docking study performed using the SwissDock platform. (A) Best energy‐ranked binding pose between meroxest (**7**) and PKCι is shown, based on the AC Score. The yellow arrow indicates the position of the ligand in the carboxy‐terminal lobe of the protein. (B, C) Surface representation of PKCι showing the interaction of meroxest (**7**) with residue Phe423 (B) and Gly398 (C) through hydrogen bonds.

Based on the kinase screening results, we next performed *in silico* docking analyses to investigate whether meroxest (**7**) could interact with the catalytic site of PKCι. The crystal structure of PKCι obtained from the Protein Data Bank (PDB ID: 1ZRZ) was used as the target in docking calculations with Attracting Cavities as implemented in SwissDock (Röhrig et al. 2023) (Zoete et al. 2016) (Grosdidier et al. 2011) (Messerschmidt et al. 2005). Docking simulations generated 109 potential binding poses, which were grouped into 49 clusters according to the AC Score and SwissParam Score (Table S2). The most favorable clusters were located near the catalytic site, with the methyl ester moiety of meroxest (**7**) oriented within the ATP‐binding cleft (Figure 3A, Figure S1).

Structural inspection in UCSF ChimeraX revealed a hydrogen bond between the carbonyl group of meroxest (**7**) and Gly398 (distance: 2.053 Å), located within the activation loop of the catalytic site (Figure 3C) (Soriano et al. 2016; Messerschmidt et al. 2005). Hydrogen bonds between ATP‐competitive kinase inhibitors and backbone atoms in or near the hinge region are a well‐documented feature of kinase inhibition, as they anchor the ligand within the adenine pocket and contribute to stabilization of the inhibitor‐protein complex (Zhang et al. 2009). A second hydrogen bond was detected with Phe423 (distance: 2.425 Å), located within the ATP‐binding cleft (Figure 3B). Engagement of residues in this region has been shown to contribute to selectivity among closely related kinases (Knight and Shokat 2005; Huse and Kuriyan 2002). Thus, interaction with Phe423 may help to define a distinctive binding profile for meroxest (**7**) that favors selectivity toward PKCι over other PKC isoforms. Binding at this position lies within the canonical ATP‐binding cleft described for eukaryotic protein kinases (Manning et al. 2002).

Taken together, these docking results support a plausible model in which meroxest (**7**) occupies the catalytic pocket of PKCι and may inhibit its kinase activity by engaging residues critical for ATP coordination and hinge stabilization. Thus, meroxest (**7**) may effectively suppress downstream mitogenic signaling. This proposed ATP‐competitive mechanism contrasts with inhibitors that disrupt PB1‐Par6 interactions at the regulatory PB1 domain (Fields et al. 2007), highlighting a distinct inhibitory strategy. However, this structural model should be interpreted cautiously. Docking does not establish target selectivity or functional mechanism by itself, and the present analysis does not exclude the possibility that other kinases identified in the primary screening assay, particularly Cdc7/cyclin B1, may also contribute to the biological activity of meroxest (**7**). Further validation through molecular dynamics simulations and in vitro kinase assays will be required to confirm the stability and functional relevance of the binding poses.

### 3.3 Design and Synthesis of Meroxest (7) Derivatives

After identifying PKCι as the most strongly inhibited kinase in the profiling assay and characterizing a plausible binding mode for meroxest (**7**) within the PKCι catalytic site, we sought to develop a new library of merosesquiterpene derivatives to enhance or modulate its inhibitory activity. Our strategy focused on modifying the naphthoquinone scaffold of meroxest (**7**) to examine how these structural changes influence its inhibitory capacity against PKCι as an early scaffold refinement.

#### 3.3.1 Modifications of the Quinone Moiety

**SCHEME 1:**
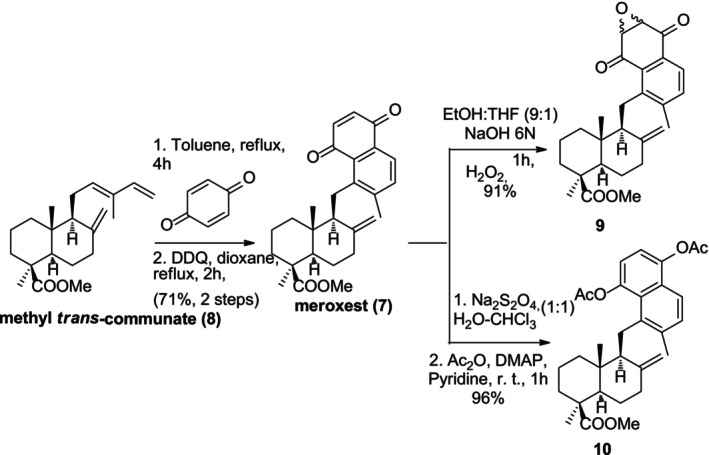
Modifications of the quinone moiety of meroxest (**7**).

First, we targeted the quinone core of meroxest (**7**) to assess its contribution to PKCι inhibition. Treatment of meroxest (**7**) with H_2_O_2_ in basic medium afforded the epoxide derivative **9** in good yield as a 1:1 mixture of diastereomers, without affecting the methyl ester group (Scheme 1). This transformation was designed to assess whether epoxidation of the quinone ring would alter the electrophilic character and potential hydrogen‐bonding pattern required for PKCι binding. In a second modification, the diacetylated derivative **10** was obtained by reduction of meroxest (**7**) with Na_2_S_2_O_4_ followed by acetylation with acetic anhydride in pyridine, introducing two acetate groups into the quinone framework. Together, these derivatives were intended to probe how alterations in the redox‐active sites and potential hydrogen‐bonding interactions affect enzyme recognition and binding affinity.

#### 3.3.2 Modifications of the Aromatic Ring

**SCHEME 2:**
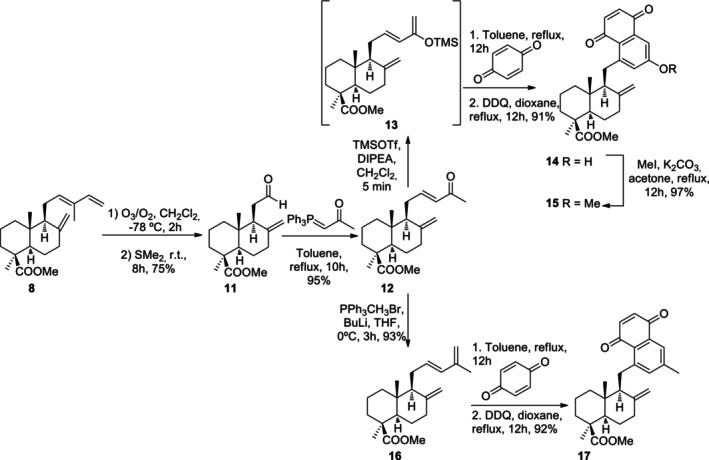
Modifications of the aromatic ring moiety of meroxest (**7**).

In parallel, we developed a new synthetic route to introduce structural changes in the aromatic ring adjacent to the quinone moiety, using methyl *trans*‐communate (**8**) as the starting material (Scheme 2). Reductive ozonolysis of **8** afforded aldehyde **11** in good yield, following a methodology developed in our group (Chayboun et al. 2015) which, after treatment with 1‐(triphenylphosphoranylidene)‐2‐propanone in refluxing toluene, gave the *α*,*β*‐unsaturated ketone **12** in excellent yield. Afterward, ketone **12** was converted into diene **13** using trimethylsilyl trifluoromethanesulfonate and *N,N*‐diisopropylethylamine. Diene **13** allowed access to derivatives **14** and **15**, bearing an oxygenated substituent on the naphthoquinone ring (hydroxy or methoxy substituent, respectively). Thus, Diels‐Alder cycloaddition of diene **13** with 1,4‐benzoquinone followed by aromatization with DDQ afforded hydroxyquinone **14**, which after methylation with methyl iodide and potassium carbonate under reflux gave methoxyquinone **15**. These modifications were designed to explore the influence of additional hydrogen‐bond donors/acceptors and increased polarity on PKCι binding.

Furthermore, a new derivative **17** was synthesized from *α*,*β*‐unsaturated ketone **12**. Treatment of **12** with methyltriphenylphosphonium bromide in the presence of *n‐*butyllithium yielded diene **16**, which underwent Diels‐Alder cycloaddition with 1,4‐benzoquinone followed by aromatization with DDQ, affording the naphthoquinone **17** in excellent yield. This sequence relocated the methyl substituent on the aromatic ring of meroxest (**7**), allowing us to evaluate how shifting the position of this substituent affects steric or electronic interactions in the PKCι catalytic site.

#### 3.3.3 Removing the Methyl Group

**SCHEME 3:**
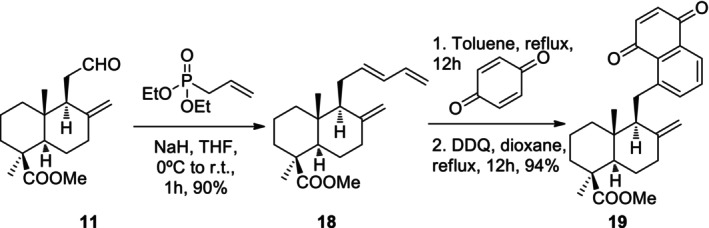
Synthesis of meroxest derivative **19** lacking the methyl substituent on the naphthoquinone moiety.

Finally, to obtain a derivative lacking the methyl group on the naphthoquinone ring of meroxest (**7**), we followed the synthetic route depicted in Scheme 3, again starting from aldehyde **11**. After treating aldehyde **11** with diethyl allylphosphonate and sodium hydride under reflux in THF, we obtained the diene **18** in good yield. Subsequent Diels‐Alder cycloaddition of 1,4‐benzoquinone with diene **18**, followed by aromatization with DDQ, provided derivative **19**, a meroxest analogue devoid of the methyl substituent on the naphthoquinone ring. All newly synthesized compounds were fully characterized by NMR, IR, and HRMS (see Supporting Information).

### 3.4 Preliminary PKCι‐Focused Activity Comparison of Meroxest Derivatives

**FIGURE 4:**
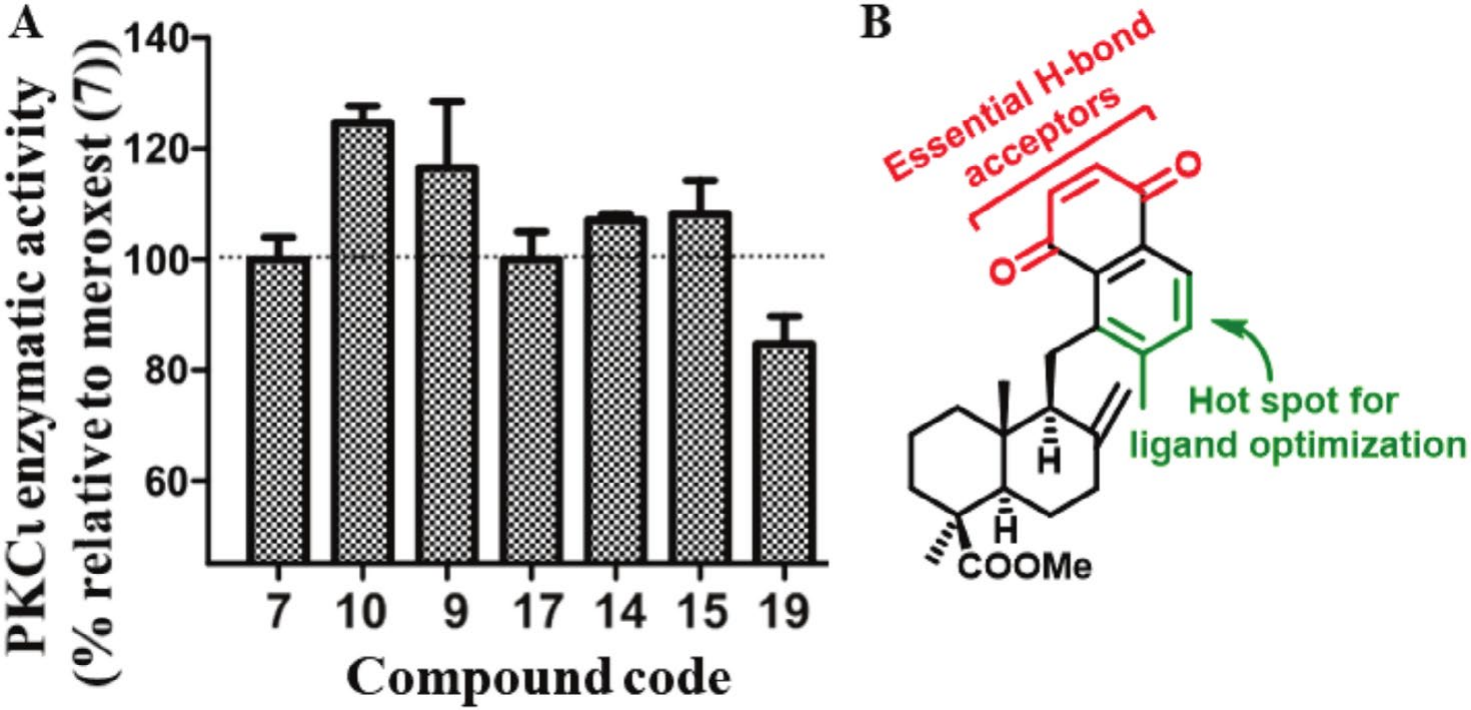
(A) PKCι enzymatic activity reported relative to meroxest (**7**) (set at 100%) in the presence of the newly synthesized library of derivatives at 10 μM. Lower values indicate stronger inhibition. (B) Position for affinity‐improving modifications on meroxest (**7**).

Having completed the library of meroxest derivatives, we next evaluated their PKCι inhibitory activity at a concentration of 10 μM using the Eurofins KinaseProfiler LeadHunter technology (Figure 4A). In the present study, PKCι was selected as the primary follow‐up target from the kinase profiling experiment because it was the most strongly inhibited kinase in the 410‐member panel and because structural information was available for docking‐guided analysis. Accordingly, the results shown in Figure 4A should be interpreted as a first PKCι‐focused comparison within the analogue series rather than as a comprehensive characterization of kinase selectivity across the scaffold.

Under these assay conditions, meroxest (**7**) reduced PKCι enzymatic activity to 52% of control levels. Overall, the tested analogues produced only limited changes relative to the parent compound. As anticipated, derivatives **9** and **10** exhibited weaker effects than meroxest (**7**) on the inhibitory potency, confirming that the integrity of the quinone moiety is crucial for PKCι binding. In contrast, shifting the position of the methyl group on the aromatic ring (derivative **17**) did not produce a significant alteration in inhibitory capacity relative to meroxest (***7***), suggesting that this particular substituent position is less critical for PKCι recognition.

By comparison, replacing the methyl group on the naphthoquinone ring with an oxygenated substituent (hydroxy, **14**; or methoxy, **15**) led to a decrease in PKCι inhibitory activity relative to meroxest (**7**). This result implies that introducing polar functionalities in this position can disrupt key interactions or induce steric constraints that prevent optimal occupation of the PKCι catalytic site. Notably, the derivative lacking the methyl substituent on the naphthoquinone ring (**19**) exhibited the most favorable inhibition profile in this PKCι‐focused comparison assay, suggesting that minimal steric bulk in that position could favor optimal binding.

Overall, these findings reinforce the critical role of the quinone moiety in PKCι inhibition and demonstrate how specific modifications, whether in the core naphthoquinone unit or in the peripheral aromatic ring, can preserve, abolish, or improve PKCι inhibitory activity (Figure 4B). In particular, derivative **19** emerged as a promising lead for further optimization, potentially opening new avenues for the design of effective PKCι‐targeted therapies, especially in indications where PKCι is a known driver of oncogenic signaling.

### 3.5 Molecular Docking Analysis of Compound 19

**FIGURE 5:**
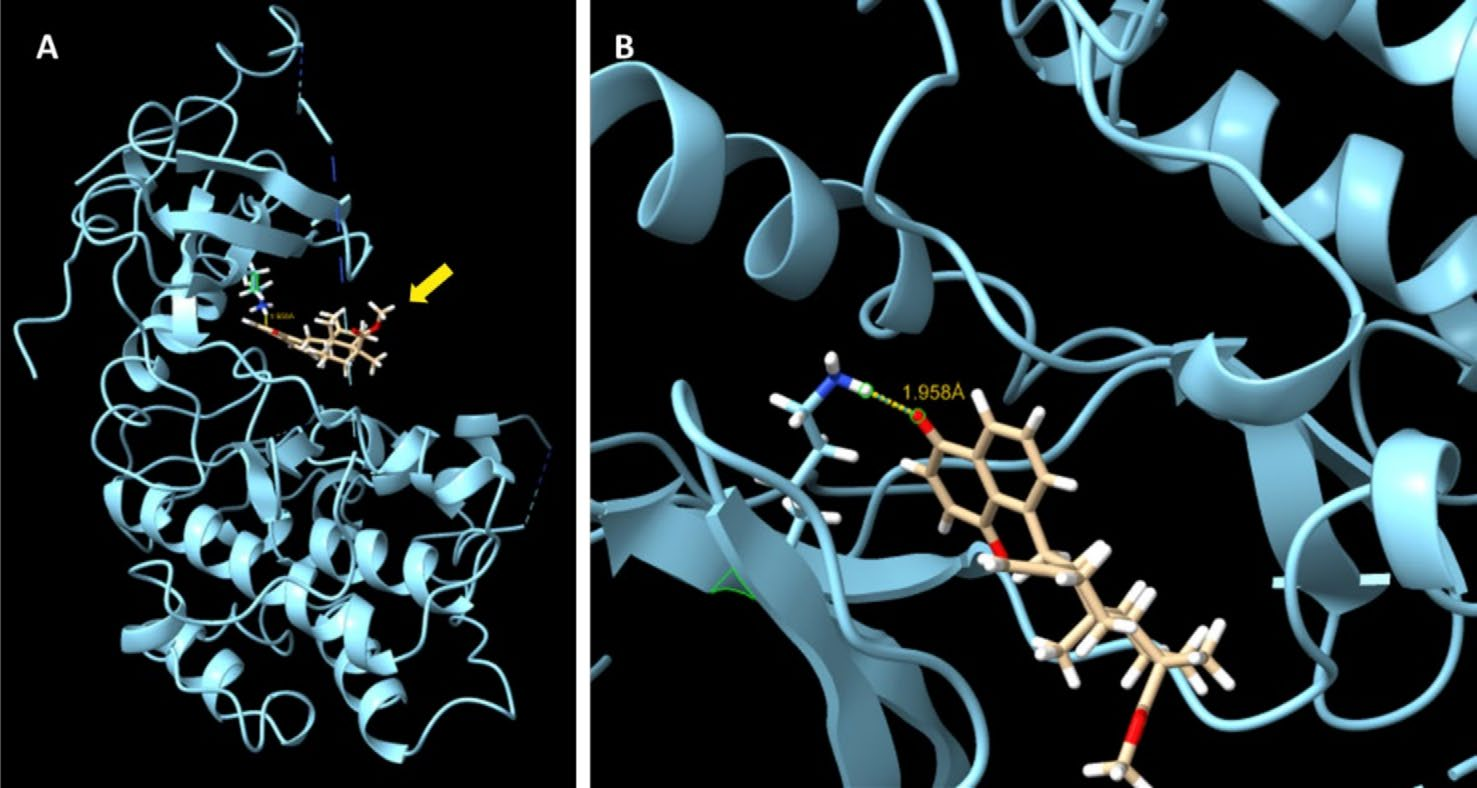
Graphical results of the molecular docking study performed using the SwissDock platform. (A) Best energy‐ranked binding pose between compound **19** and PKCι based on the AC Score. (B) Surface representation of PKCι showing the interaction between Lys274 and the quinone moiety of compound **19** through a hydrogen bond.

To explore a possible structural basis for the activity profile of the most informative analogue in the series, we performed docking analysis of compound 19 using the same SwissDock/PKCι framework applied to meroxest (7) (Figure 5, Table S3, Figure S2). Docking analyses indicate that the newly designed compound **19** can adopt a binding pose in which it forms a hydrogen bond with Lys274 (distance: 1.958 Å) within the catalytic site of PKCι (Figure 5, Table S3, Figure S2). Consistently, previous modeling of PKCι inhibitors has also identified Lys274 as a key hydrogen‐bonding residue (Kwiatkowski et al. 2018). This interaction was not observed with the parent inhibitor meroxest (**7**) and may therefore contribute to the somewhat more favorable PKCι‐focused profile observed for compound 19 under the assay conditions used. Although Lys274 is located in the ATP‐binding region and certain mutations at this position (e.g., Lys274Arg) are tolerated without complete loss of catalytic activity (Spitaler et al. 2000), engagement of this residue by compound **19** may help stabilize the ligand within the active site. In this context, the interaction with Lys274 provides a plausible structural rationale for the modest improvement observed for compound **19** relative to meroxest (**7**) in the biochemical assay.

Nevertheless, this interpretation should be considered with appropriate caution. While the docking analysis suggests a potential role for Lys274 engagement, it does not establish it as the sole determinant of activity, nor does it confirm improved kinase selectivity. In addition, as the analogue series was not evaluated against Cdc7/cyclin B1 or in cell‐based target‐engagement assays, the broader functional implications of this binding model remain to be explored. Thus, the docking results for compound **19** are best regarded as a structural hypothesis that may guide future analogue design, rather than as definitive evidence of an optimized PKCι inhibitor.

## 4 Conclusions

In this study, meroxest (**7**) showed its strongest inhibitory effect in a 410‐member kinase panel against PKCι, reducing enzymatic activity to 52% of control at 10 μM. While most other kinases, including related PKC isoforms, were only weakly affected, additional inhibition of Cdc7/cyclin B1 was also observed, suggesting that the mechanism of action may not be exclusively PKCι‐driven.

Docking analyses supported a plausible ATP‐competitive binding mode of meroxest (**7**) within the catalytic pocket of PKCι, involving interactions with key residues such as Phe423 and Gly398. Complementary SAR studies using a focused library of meroxest‐derived analogues highlighted the importance of the naphthoquinone core and the aromatic framework for activity. Although most structural modifications resulted in only modest changes in inhibitory potency under the conditions tested, compound **19**, lacking the methyl substituent on the naphthoquinone ring, displayed a comparatively more favorable PKCι‐oriented profile and may represent a useful starting point for further scaffold refinement.

Overall, these results support PKCι as a relevant molecular target within a broader multi‐kinase interaction profile for meroxest (**7**) and provide an initial candidate for the rational design of improved analogues. At the same time, the magnitude of inhibition observed remains moderate; the analogue series has not yet been evaluated against Cdc7/cyclin B1 or in cell‐based target‐engagement assays, and the broader functional implications of the proposed binding model remain to be established. Accordingly, the present findings are best viewed as defining a PKCι‐prioritized mechanistic direction and a basis for future optimization, rather than as definitive evidence of a highly potent or selective PKCι inhibitor series. Further biochemical and cellular studies will be required to clarify the relative contribution of PKCι, Cdc7/cyclin B1, and other potential targets to the biological activity of this scaffold.

## Author Contributions

**Rachid Chahboun:** funding acquisition, conceptualization, investigation, methodology, writing – review and editing, project administration, resources, supervision, validation, data curation. **Ángel Bueno:** formal analysis, data curation, investigation, visualization, validation. **Nuria Mut‐Salud:** validation, visualization, data curation, investigation, formal analysis. **Antonio Fernández:** conceptualization, methodology, investigation, funding acquisition, resources, supervision, writing – original draft, validation, formal analysis. **Ramón Alvarez‐Manzaneda:** investigation, methodology. **Fernando Rodríguez‐Serrano:** conceptualization, funding acquisition, writing – review and editing, formal analysis, supervision, investigation, methodology, data curation. **Enrique Alvarez‐Manzaneda:** conceptualization, project administration.

## Funding

This work was supported by Consejería de Conocimiento, Investigación y Universidad, Junta de Andalucía, C‐EXP‐053‐UGR23.

## Conflicts of Interest

The authors declare no conflicts of interest.

## Supporting information


**Table S1:** Kinase profiling assay of meroxest (**7**) covering 410 protein kinases using Eurofins KinaseProfiler technology at a concentration of 10 μM. Values are reported as residual enzyme activity relative to control.
**Table S2:** Results of the best potential binding poses organized into 49 clusters according to the AC Score between meroxest (7) and PKC iota, according to the analysis performed using the SwissDock platform.
**Table S3:** Results of the best potential binding poses organized into 49 clusters according to the AC Score between compound **19** and PKC iota, according to the analysis performed using the SwissDock platform.
**Figure S1:** Graphical results of the molecular docking analysis performed with SwissDock. Visualization of the interaction between PKC iota and meroxest (**7**).
**Figure S2:** Graphical results of the molecular docking analysis performed with SwissDock. Visualization of the interaction between PKC iota and compound **19**.

## Data Availability

Additional data are available from the corresponding author upon reasonable request.
