# Supplementary material for: Design, Optimization and Mechanistic Insights Into Terpenoid‐Derived Inhibitors of Protein Kinase C Iota
Source: Chem Biol Drug Des. 2026 Jun 26;107(6):e70348. doi: 10.1111/cbdd.70348 (PMC13307258; doi:10.1111/cbdd.70348)
Supplement: Supplementary file 1 — Table S1: Kinase profiling assay of meroxest (7) covering 410 protein kinases using Eurofins KinaseProfiler technology at a concentration of 10 μM. Values are reported as residual enzyme activity relative to control. Table S2: Results of the best potential binding poses organized into 49 clusters according to the AC Score between meroxest (7) and PKC iota, according to the analysis performed using the SwissDock platform. Table S3: Results of the best potential binding poses organized into 49 clusters according to the AC Score between compound 19 and PKC iota, according to the analysis performed using the SwissDock platform. Figure S1: Graphical results of the molecular docking analysis performed with SwissDock. Visualization of the interaction between PKC iota and meroxest (7). Figure S2: Graphical results of the molecular docking analysis performed with SwissDock. Visualization of the interaction between PKC iota and compound 19. [file CBDD-107-e70348-s001.docx]

**Supporting Information**

**Design, Optimization and Mechanistic Insights into Terpenoid-Derived Inhibitors of Protein Kinase C Iota**

**Rachid Chahboun^1,#^, Fernando Rodríguez-Serrano^2,3,#^, Ángel Bueno^1^, Nuria Mut-Salud^2^,** **Ramón Alvarez-Manzaneda^4^, Enrique Alvarez-Manzaneda^1^, Antonio Fernández^1*^**

^1^Department of Organic Chemistry, Faculty of Sciences, Institute of Biotechnology, University of Granada, Granada 18071, Spain

^2^Biopathology and Regenerative Medicine Institute (IBIMER), University of Granada, Granada 18016, Spain

^3^Instituto de Investigación Biosanitaria de Granada (ibs.GRANADA), Granada, 18012, Spain

^4^Area of Organic Chemistry, Department of Chemistry and Physics, University of Almería, 04120 Almería, Spain

***Correspondence**: Antonio Fernández ([ajfvargas@ugr.es](mailto:ajfvargas@ugr.es))

#Both authors contributed equally to this work

**Table of Contents**

1. **Kinase profiling assay of meroxest (7)**
2. **SwissDock results**
3. **Synthetic procedures and characterization data of the synthesized compounds**
4. **^1^H NMR, ^13^C NMR and HRMS spectra for new compounds**

Table S1. Kinase profiling assay of meroxest (**7**) covering 410 protein kinases using Eurofins KinaseProfiler™ technology at a concentration of 10 µM. Values are reported as residual enzyme activity relative to control.

| Kinase | Residual Enzyme Activity (% of control; Meroxest 10 μM) |
| --- | --- |
| Abl(h) | 95 |
| Abl(m) | 97 |
| Abl (H396P) (h) | 117 |
| Abl (M351T)(h) | 118 |
| Abl (Q252H) (h) | 100 |
| Abl(T315I)(h) | 107 |
| Abl(Y253F)(h) | 98 |
| ACK1(h) | 100 |
| ACTR2(h) | 107 |
| ALK(h) | 121 |
| ALK1(h) | 138 |
| ALK2(h) | 113 |
| ALK4(h) | 103 |
| ALK6(h) | 94 |
| Arg(h) | 134 |
| AMPKα1(h) | 91 |
| AMPKα2(h) | 91 |
| A-Raf(h) | 100 |
| Arg(m) | 115 |
| ARK5(h) | 106 |
| ASK1(h) | 100 |
| Aurora-A(h) | 82 |
| Aurora-B(h) | 76 |
| Aurora-C(h) | 101 |
| Axl(h) | 97 |
| Blk(h) | 71 |
| Blk(m) | 89 |
| BMPR2(h) | 106 |
| Bmx(h) | 107 |
| BRK(h) | 106 |
| BrSK1(h) | 93 |
| BrSK2(h) | 99 |
| BTK(h) | 107 |
| BTK(R28H)(h) | 109 |
| B-Raf(h) | 103 |
| B-Raf(V599E)(h) | 98 |
| CaMKI(h) | 95 |
| CaMKIß(h) | 90 |
| CaMKIγ(h) | 96 |
| CaMKIIα(h) | 96 |
| CaMKIIβ(h) | 88 |
| CaMKIIγ(h) | 99 |
| CaMKIδ(h) | 92 |
| CaMKIIδ(h) | 108 |
| CaMKIV(h) | 85 |
| CaMKK1(h) | 95 |
| CaMKK2(h) | 108 |
| Cdc7/cyclinB1(h) | 63 |
| CDK1/cyclinB(h) | 84 |
| CDK2/cyclinA(h) | 117 |
| CDK2/cyclinE(h) | 90 |
| CDK3/cyclinE(h) | 104 |
| CDK4/cyclinD3(h) | 101 |
| CDK5/p25(h) | 100 |
| CDK5/p35(h) | 107 |
| CDK6/cyclinD3(h) | 97 |
| CDK7/cyclinH/MAT1(h) | 125 |
| CDK9/cyclin T1(h) | 98 |
| CDK12/cyclinK(h) | 98 |
| CDK13/cyclinK(h) | 107 |
| CDK14/cyclinY(h) | 99 |
| CDK18/cyclinY(h) | 120 |
| CDKL1(h) | 106 |
| CDKL2(h) | 101 |
| CDKL3(h) | 86 |
| CDKL4(h) | 109 |
| ChaK1(h) | 102 |
| CHK1(h) | 121 |
| CHK2(h) | 81 |
| CHK2(I157T)(h) | 85 |
| CHK2(R145W)(h) | 87 |
| CK1γ1(h) | 90 |
| CK1γ2(h) | 100 |
| CK1γ3(h) | 116 |
| CK1δ(h) | 108 |
| CK1(y) | 103 |
| CK2(h) | 107 |
| CK2α1(h) | 98 |
| CK2α2(h) | 105 |
| CLIK1(h) | 117 |
| CLK1(h) | 122 |
| CLK2(h) | 88 |
| CLK3(h) | 100 |
| CLK4(h) | 98 |
| cKit(h) | 103 |
| cKit(D816V)(h) | 100 |
| cKit(D816H)(h) | 108 |
| cKit(V560G)(h) | 108 |
| cKit(V654A)(h) | 97 |
| CSK(h) | 102 |
| c-RAF(h) | 97 |
| cSRC(h) | 96 |
| DAPK1(h) | 85 |
| DAPK2(h) | 85 |
| DCAMKL2(h) | 106 |
| DCAMKL3(h) | 95 |
| DDR1(h) | 107 |
| DDR2(h) | 107 |
| DMPK(h) | 104 |
| DRAK1(h) | 116 |
| DRAK2(h) | 93 |
| DYRK1A(h) | 109 |
| DYRK1B(h) | 99 |
| DYRK2(h) | 100 |
| DYRK3(h) | 102 |
| eEF-2K(h) | 95 |
| EGFR(h) | 99 |
| EGFR(L858R)(h) | 130 |
| EGFR(L861Q)(h) | 110 |
| EGFR(T790M)(h) | 95 |
| EGFR(T790M,L858R)(h) | 111 |
| EphA1(h) | 90 |
| EphA2(h) | 101 |
| EphA3(h) | 107 |
| EphA4(h) | 114 |
| EphA5(h) | 110 |
| EphA7(h) | 104 |
| EphA8(h) | 104 |
| EphB2(h) | 102 |
| EphB1(h) | 115 |
| EphB3(h) | 96 |
| EphB4(h) | 101 |
| ErbB2(h) | 120 |
| ErbB4(h) | 105 |
| FAK(h) | 102 |
| Fer(h) | 103 |
| Fes(h) | 111 |
| FGFR1(h) | 99 |
| FGFR1(V561M)(h) | 103 |
| FGFR2(h) | 89 |
| FGFR2(N549H)(h) | 105 |
| FGFR3(h) | 109 |
| FGFR4(h) | 101 |
| Fgr(h) | 110 |
| Flt1(h) | 102 |
| Flt3(D835Y)(h) | 128 |
| Flt3(h) | 100 |
| Flt4(h) | 90 |
| Fms(h) | 86 |
| Fms(Y969C)(h) | 107 |
| Fyn(h) | 112 |
| GCK(h) | 95 |
| GCN2(h) | 105 |
| GRK1(h) | 106 |
| GRK2(h) | 100 |
| GRK3(h) | 93 |
| GRK5(h) | 95 |
| GRK6(h) | 98 |
| GRK7(h) | 84 |
| GSK3α(h) | 96 |
| GSK3β(h) | 100 |
| Haspin(h) | 106 |
| Hck(h) | 96 |
| Hck(h) activated | 96 |
| HIPK1(h) | 92 |
| HIPK2(h) | 100 |
| HIPK3(h) | 83 |
| HIPK4(h) | 105 |
| HPK1(h) | 97 |
| HRI(h) | 115 |
| ICK(h) | 110 |
| IGF-1R(h) | 138 |
| IGF-1R(h), activated | 112 |
| IKKα(h) | 109 |
| IKKβ(h) | 99 |
| IKKε(h) | 99 |
| IR(h) | 140 |
| IR(h), activated | 90 |
| IRE1(h) | 98 |
| IRR(h) | 107 |
| IRAK1(h) | 99 |
| IRAK4(h) | 93 |
| Itk(h) | 99 |
| JAK1(h) | 110 |
| JAK2(h) | 115 |
| JAK3(h) | 84 |
| JNK1α1(h) | 96 |
| JNK2α2(h) | 85 |
| JNK3(h) | 91 |
| KDR(h) | 107 |
| Lck(h) | 173 |
| Lck(h) activated | 100 |
| LIMK1(h) | 95 |
| LIMK2(h) | 102 |
| LKB1(h) | 96 |
| LOK(h) | 94 |
| Lyn(h) | 92 |
| Lyn(m) | 93 |
| LRRK2(h) | 101 |
| LTK(h) | 114 |
| MAK(h) | 104 |
| MAPK1(h) | 95 |
| MAPK2(h) | 101 |
| MAPK2(m) | 99 |
| MAP4K3(h) | 102 |
| MAP4K4(h) | 102 |
| MAP4K5(h) | 90 |
| MAPKAP-K2(h) | 101 |
| MAPKAP-K3(h) | 98 |
| MEK1(h) | 103 |
| MEK2(h) | 110 |
| MARK1(h) | 107 |
| MARK3(h) | 95 |
| MARK4(h) | 99 |
| MEKK2(h) | 100 |
| MEKK3(h) | 113 |
| MELK(h) | 83 |
| Mer(h) | 106 |
| Met(h) | 121 |
| Met(D1246H)(h) | 139 |
| Met(D1246N)(h) | 146 |
| Met(M1268T)(h) | 133 |
| Met(Y1248C)(h) | 134 |
| Met(Y1248D)(h) | 119 |
| Met(Y1248H)(h) | 135 |
| MINK(h) | 91 |
| MKK4(m) | 96 |
| MKK6(h) | 107 |
| MKK7β(h) | 93 |
| MLCK(h) | 94 |
| MLK1(h) | 103 |
| MLK2(h) | 115 |
| Mnk2(h) | 97 |
| MOK(h) | 101 |
| MRCKα(h) | 114 |
| MRCKβ(h) | 102 |
| MSK1(h) | 107 |
| MSK2(h) | 103 |
| MSSK1(h) | 102 |
| MST1(h) | 107 |
| MST2(h) | 99 |
| MST3(h) | 90 |
| MST4(h) | 98 |
| mTOR(h) | 116 |
| mTOR/FKBP12(h) | 100 |
| MuSK(h) | 109 |
| MYLK2(h) | 98 |
| MYO3B(h) | 106 |
| NDR2(h) | 96 |
| NEK1(h) | 103 |
| NEK2(h) | 121 |
| NEK4(h) | 98 |
| NEK3(h) | 77 |
| NEK6(h) | 94 |
| NEK7(h) | 128 |
| NEK9(h) | 128 |
| NIM1(h) | 116 |
| NEK11(h) | 104 |
| NLK(h) | 107 |
| NUAK2(h) | 118 |
| p70S6K(h) | 82 |
| PAK1(h) | 108 |
| PAK2(h) | 98 |
| PAK4(h) | 98 |
| PAK3(h) | 96 |
| PAK5(h) | 104 |
| PAK6(h) | 112 |
| PAR-1Bα(h) | 105 |
| PASK(h) | 114 |
| PEK(h) | 92 |
| PDGFRα(h) | 115 |
| PDGFRα(D842V)(h) | 99 |
| PDGFRα(V561D)(h) | 109 |
| PDGFRβ(h) | 127 |
| PDHK4(h) | 91 |
| PDK1(h) | 94 |
| PhKγ1(h) | 105 |
| PhKγ2(h) | 109 |
| Pim-1(h) | 85 |
| Pim-2(h) | 83 |
| Pim-3(h) | 100 |
| PKA(h) | 88 |
| PKAcβ(h) | 119 |
| PKBα(h) | 103 |
| PKBβ(h) | 102 |
| PKBγ(h) | 104 |
| PKCα(h) | 94 |
| PKCβI(h) | 99 |
| PKCβII(h) | 97 |
| PKCγ(h) | 124 |
| PKCδ(h) | 104 |
| PKCε(h) | 113 |
| PKCη(h) | 103 |
| PKCι(h) | 52 |
| PKCμ(h) | 101 |
| PKCθ(h) | 100 |
| PKCζ(h) | 106 |
| PKD2(h) | 92 |
| PKD3(h) | 106 |
| PKG1α(h) | 101 |
| PKG1β(h) | 120 |
| PKR(h) | 103 |
| Plk1(h) | 93 |
| Plk3(h) | 110 |
| Plk4(h) | 100 |
| PRAK(h) | 89 |
| PRKG2(h) | 104 |
| PRK1(h) | 101 |
| PRK2(h) | 105 |
| PrKX(h) | 99 |
| PRP4(h) | 83 |
| PTK5(h) | 98 |
| Pyk2(h) | 90 |
| Ret(h) | 102 |
| Ret (V804L)(h) | 124 |
| Ret(V804M)(h) | 144 |
| RIPK1(h) | 113 |
| RIPK2(h) | 101 |
| ROCK-I(h) | 108 |
| ROCK-II(h) | 110 |
| ROCK-II(r) | 102 |
| Ron(h) | 103 |
| Ros(h) | 105 |
| Rse(h) | 124 |
| Rsk1(h) | 113 |
| Rsk1(r) | 134 |
| Rsk2(h) | 114 |
| Rsk3(h) | 103 |
| Rsk4(h) | 121 |
| SAPK2a(h) | 97 |
| SAPK2a(T106M)(h) | 110 |
| SAPK2b(h) | 89 |
| SAPK3(h) | 101 |
| SAPK4(h) | 98 |
| SBK1(h) | 107 |
| SGK(h) | 108 |
| SGK2(h) | 92 |
| SGK3(h) | 107 |
| SIK(h) | 108 |
| SIK2(h) | 107 |
| SIK3(h) | 104 |
| SLK(h) | 87 |
| Snk(h) | 101 |
| SNRK(h) | 99 |
| Src(1-530)(h) | 103 |
| Src(T341M)(h) | 106 |
| SRPK1(h) | 79 |
| SRPK2(h) | 96 |
| STK16(h) | 99 |
| STK25(h) | 91 |
| STK32A(h) | 106 |
| STK32B(h) | 81 |
| STK32C(h) | 95 |
| STK33(h) | 93 |
| Syk(h) | 138 |
| TAF1L(h) | 107 |
| TAK1(h) | 97 |
| TAO1(h) | 95 |
| TAO2(h) | 100 |
| TAO3(h) | 94 |
| TBK1(h) | 106 |
| Tec(h) activated | 95 |
| TGFBR1(h) | 103 |
| TGFBR2(h) | 91 |
| Tie2 (h) | 107 |
| Tie2(R849W)(h) | 105 |
| Tie2(Y897S)(h) | 99 |
| TLK1(h) | 106 |
| TLK2(h) | 96 |
| TNIK(h) | 100 |
| TRB2(h) | 96 |
| TrkA(h) | 69 |
| TrkB(h) | 77 |
| TrkC(h) | 99 |
| TSSK1(h) | 102 |
| TSSK2(h) | 109 |
| TSSK3(h) | 87 |
| TSSK4(h) | 111 |
| TTBK1(h) | 110 |
| TTBK2(h) | 107 |
| TTK(h) | 93 |
| Txk(h) | 107 |
| TYK2(h) | 95 |
| ULK1(h) | 99 |
| ULK2(h) | 106 |
| ULK3(h) | 94 |
| VRK1(h) | 113 |
| VRK2(h) | 100 |
| Wee1(h) | 94 |
| Wee1B(h) | 101 |
| WNK1(h) | 123 |
| WNK2(h) | 103 |
| WNK3(h) | 103 |
| Yes(h) | 98 |
| ZAK(h) | 95 |
| ZAP-70(h) | 89 |
| ZIPK(h) | 102 |
| ATM(h) | 87 |
| ATR/ATRIP(h) | 97 |
| DNA-PK(h) | 97 |
| PI3 Kinase (p110β/p85α)(h) | 92 |
| PI3 Kinase (p120γ)(h) | 93 |
| PI3 Kinase (p110δ/p85α)(h) | 95 |
| PI3 Kinase (p110α/p85α)(m) | 88 |
| PI3 Kinase (p110α/p65α)(m) | 92 |
| PI3 Kinase (p110α(E545K)/p85α)(m) | 88 |
| PI3 Kinase (p110α(H1047R)/p85α)(m) | 86 |
| PI3 Kinase (p110β/p85β)(m) | 94 |
| PI3 Kinase (p110β/p85α)(m) | 93 |
| PI3 Kinase (p110δ/p85α)(m) | 94 |
| PI3 Kinase (p110α(E542K)/p85α)(m) | 88 |
| PI3 Kinase (p110α/p85α)(h) | 90 |
| PI3 Kinase (p110α(E542K)/p85α)(h) | 90 |
| PI3 Kinase (p110α(H1047R)/p85α)(h) | 88 |
| PI3 Kinase (p110α(E545K)/p85α)(h) | 79 |
| PI3 Kinase (p110α/p65α)(h) | 85 |
| PI3KC2α(h) | 85 |
| PI3KC2γ(h) | 93 |
| PIP4K2α(h) | 100 |
| PIP5K1α(h) | 102 |
| PIP5K1γ(h) | 91 |

Table S2. Results of the best potential binding poses organized into 49 clusters according to the AC Score between meroxest (**7**) and PKC iota, according to the analysis performed using the SwissDock platform.

| Cluster number | Cluster member | AC Score | SwissParam Score |
| --- | --- | --- | --- |
| 0 | 1 | 33.744192 | -7.1187 |
| 1 | 1 | 34.333253 | -7.2401 |
| 2 | 1 | 34.380375 | -8.1085 |
| 3 | 1 | 35.234893 | -7.4309 |
| 4 | 1 | 35.904893 | -7.5487 |
| 5 | 1 | 36.928869 | -7.0227 |
| 6 | 1 | 36.957592 | -7.4897 |
| 7 | 1 | 36.966289 | -7.3767 |
| 8 | 1 | 37.189156 | -7.3371 |
| 9 | 1 | 37.299019 | -7.421 |
| 10 | 1 | 39.080394 | -6.6794 |
| 11 | 1 | 39.3356 | -6.7426 |
| 12 | 1 | 39.341963 | -6.714 |
| 13 | 1 | 39.754256 | -6.8055 |
| 14 | 1 | 40.269408 | -6.323 |
| 15 | 1 | 40.521735 | -6.937 |
| 16 | 1 | 40.891882 | -6.4338 |
| 17 | 1 | 40.978284 | -6.5622 |
| 18 | 1 | 41.07333 | -6.8162 |
| 19 | 1 | 41.295029 | -6.8398 |
| 20 | 1 | 41.487341 | -6.3354 |
| 21 | 1 | 41.535495 | -7.4717 |
| 22 | 1 | 41.944291 | -6.2632 |
| 23 | 1 | 42.332858 | -6.3008 |
| 24 | 1 | 42.449781 | -6.4012 |
| 25 | 1 | 42.473238 | -6.1514 |
| 26 | 1 | 42.508894 | -6.6227 |
| 27 | 1 | 42.696311 | -6.2809 |
| 28 | 1 | 42.739844 | -6.6911 |
| 29 | 1 | 42.955474 | -7.6849 |
| 30 | 1 | 43.566651 | -5.8965 |
| 31 | 1 | 43.901401 | -6.9475 |
| 32 | 1 | 43.917315 | -7.4694 |
| 33 | 1 | 43.934047 | -7.1642 |
| 34 | 1 | 44.098491 | -5.9459 |
| 35 | 1 | 44.174174 | -6.7286 |
| 36 | 1 | 44.358422 | -6.1785 |
| 37 | 1 | 45.08813 | -6.1233 |
| 38 | 1 | 45.363881 | -6.464 |
| 39 | 1 | 45.391083 | -7.5744 |
| 40 | 1 | 45.579747 | -6.1148 |
| 41 | 1 | 45.658802 | -5.9984 |
| 42 | 1 | 45.811618 | -6.0021 |
| 43 | 1 | 46.291368 | -6.6863 |
| 44 | 1 | 46.307861 | -6.3879 |
| 45 | 1 | 46.310051 | -5.6482 |
| 46 | 1 | 46.394967 | -6.6575 |
| 47 | 1 | 46.409948 | -6.3049 |
| 48 | 1 | 46.472538 | -5.8246 |
| 49 | 1 | 46.771172 | -6.3999 |

Table S3. Results of the best potential binding poses organized into 49 clusters according to the AC Score between compound **19** and PKC iota, according to the analysis performed using the SwissDock platform.

| Cluster number | Cluster member | AC Score | SwissParam Score |
| --- | --- | --- | --- |
| 0 | 1 | 25.793046 | -7.5989 |
| 1 | 1 | 28.80731 | -7.4178 |
| 2 | 1 | 29.242345 | -8.0765 |
| 3 | 1 | 32.826899 | -7.9012 |
| 4 | 1 | 33.370483 | -7.2978 |
| 5 | 1 | 37.037198 | -7.8781 |
| 6 | 1 | 37.205373 | -7.521 |
| 7 | 1 | 37.724984 | -6.543 |
| 8 | 1 | 38.144095 | -7.3654 |
| 9 | 1 | 38.30368 | -7.4153 |
| 10 | 1 | 38.386985 | -7.1667 |
| 11 | 1 | 38.853143 | -7.7231 |
| 12 | 1 | 39.483232 | -7.4227 |
| 13 | 1 | 39.53531 | -7.2824 |
| 14 | 1 | 39.666452 | -7.5552 |
| 15 | 1 | 39.895512 | -7.068 |
| 16 | 1 | 40.175861 | -6.9143 |
| 17 | 1 | 41.274136 | -6.4658 |
| 18 | 1 | 41.287906 | -6.8675 |
| 19 | 1 | 41.352268 | -7.236 |
| 20 | 1 | 41.752089 | -7.7401 |
| 21 | 1 | 41.83775 | -7.3297 |
| 22 | 1 | 41.947785 | -6.9761 |
| 23 | 1 | 41.985517 | -7.473 |
| 24 | 1 | 42.076485 | -7.1659 |
| 25 | 1 | 42.091927 | -6.8224 |
| 26 | 1 | 42.145851 | -6.5261 |
| 27 | 1 | 42.861573 | -6.6183 |
| 28 | 1 | 42.969186 | -7.8747 |
| 29 | 1 | 43.652713 | -6.0992 |
| 30 | 1 | 43.829224 | -6.348 |
| 31 | 1 | 43.946112 | -6.2561 |
| 32 | 1 | 44.358997 | -7.2669 |
| 33 | 1 | 44.456438 | -6.249 |
| 34 | 1 | 44.574132 | -6.4257 |
| 35 | 1 | 45.108093 | -6.3659 |
| 36 | 1 | 45.203741 | -6.1683 |
| 37 | 1 | 45.235546 | -6.4798 |
| 38 | 1 | 45.238825 | -8.0465 |
| 39 | 1 | 45.242038 | -6.2855 |
| 40 | 1 | 45.350755 | -6.3681 |
| 41 | 1 | 45.579152 | -6.3451 |
| 42 | 1 | 45.585965 | -6.2636 |
| 43 | 1 | 45.979591 | -6.7231 |
| 44 | 1 | 46.494631 | -5.7688 |
| 45 | 1 | 46.528084 | -6.4967 |
| 46 | 1 | 46.613424 | -5.6445 |
| 47 | 1 | 46.991636 | -7.0639 |
| 48 | 1 | 47.093006 | -6.2549 |
| 49 | 1 | 47.201278 | -6.3071 |

Figure S1. Graphical results of the molecular docking analysis performed with SwissDock. Visualization of the interaction between PKC iota and meroxest (**7**).


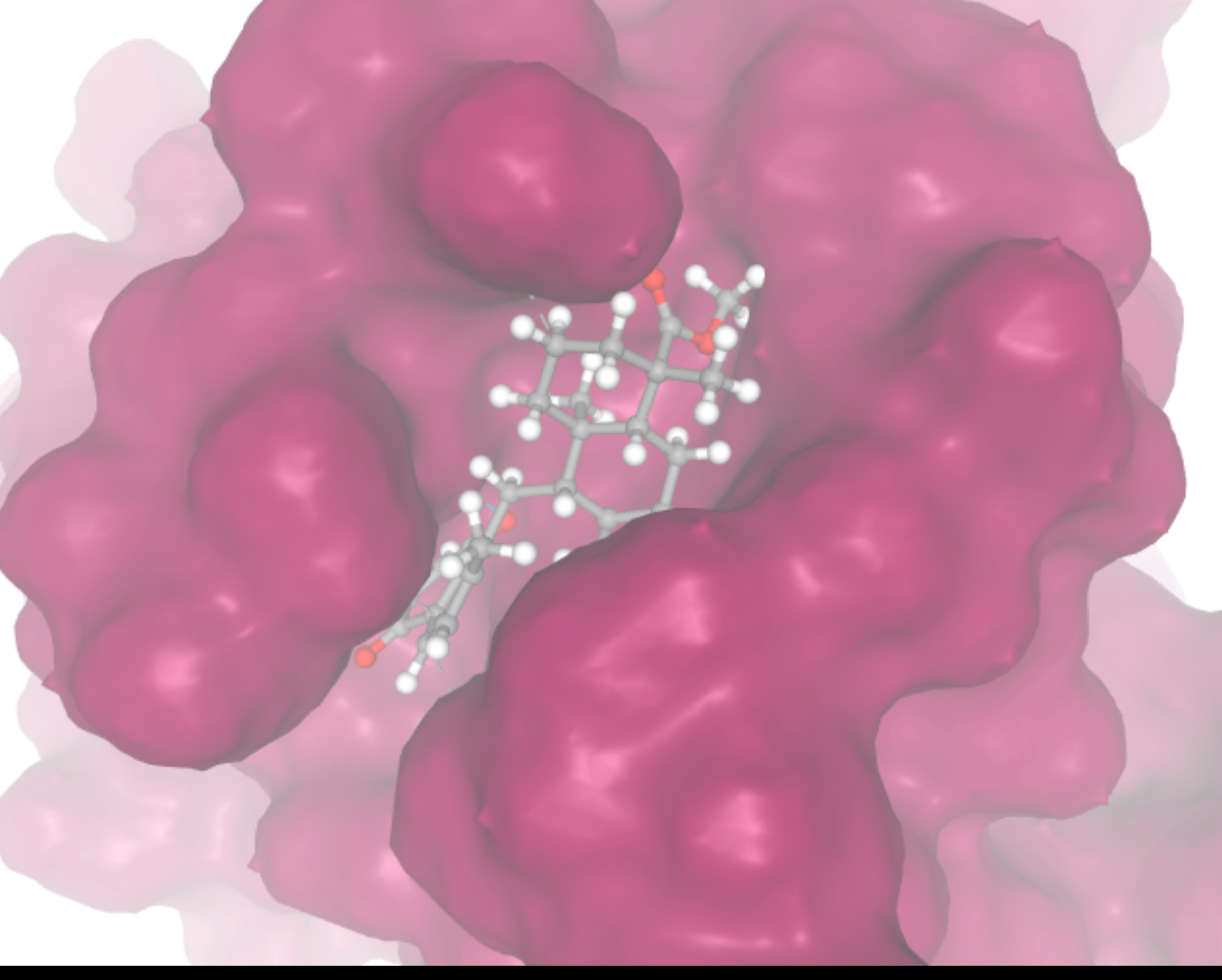


Figure S2. Graphical results of the molecular docking analysis performed with SwissDock. Visualization of the interaction between PKC iota and compound **19**.


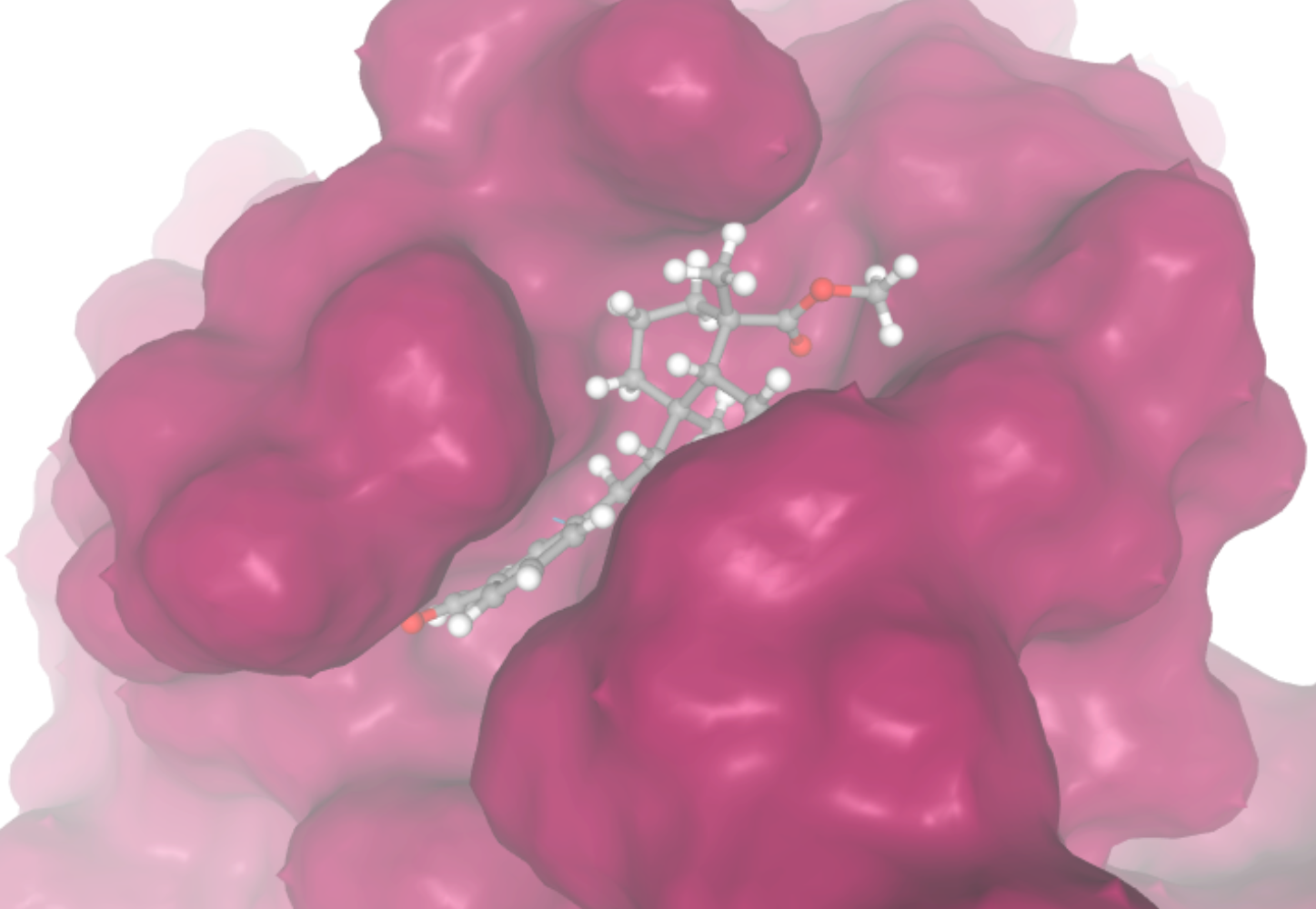


**Synthetic procedures and characterization data of the synthesized compounds**

***(1S,4aR,5S,8aR)-methyl 1,4a-dimethyl-5-((4-methyl-2,7-dioxo-1a,2,7,7a-tetrahydronaphtho[2,3-b]oxiren-3-yl)methyl)-6-methylenedecahydronaphthalene-1-carboxylate (9)***

To a stirred solution of compound **7** (250 mg, 0.60 mmol) in ethanol:THF (10 mL, 9:1) were added NaOH (1 mL, 6.0 M in EtOH) and H_2_O_2_ (0.15 mL, 30% in H_2_O) successively at room temperature. The reaction mixture was vigorously stirred at r.t. until full consumption of the starting material, observed by TLC. The solvent was removed under reduced pressure and the mixture was diluted with ether (30 mL) and washed with water (10 mL), brine (15 mL) and the organic layer was dried over Na_2_SO_4_. The solvent was removed under vacuum and the crude product was purified by silica gel chromatography (eluting with Hexane/ethyl acetate = 8/2) to yield 236 mg of **9** (91 %) as a white solid:

[**α]_D_^25^=** +85.0 (c 0.2 g/100 mL, CHCl_3_). ^1^H NMR (500 MHz, CDCl3) δ 7.69 (d, *J* = 7.8 Hz, 1H), 7.42 (d, *J* = 7.8 Hz, 1H), 4.77 (s, 1H), 4.48 (s, 1H), 4.06 (d, *J* = 4.4 Hz, 1H), 4.00 (d, *J* = 4.4 Hz, 1H), 3.64 (s, 3H), 3.60 (dd, *J* = 15.0, 5.9 Hz, 1H), 2.85 (dd, *J* = 15.0, 6.6 Hz, 1H), 2.41 (s, 3H), 2.37 (m, 1H), 2.15 (dd, *J* = 13.3, 1.5 Hz, 1H), 1.98 (t, *J* = 6.2 Hz, 2H), 1.95 – 1.81 (m, 2H), 1.59 (m, 1H), 1.51 (m, 1H), 1.30 (dd, *J* = 12.2, 3.3 Hz, 2H), 1.17 (s, 3H), 1.12 – 0.96 (m, 2H), 0.66 (s, 3H). ^13^C NMR (126 MHz, CDCl_3_) δ = 195.9, 191.5, 177.6, 149.0, 146.1, 144.7, 134.9, 132.6, 130.5, 124.6, 107.0, 57.6, 56.5, 55.5, 54.5, 51.2, 44.3, 41.9, 39.3, 38.6, 38.0, 28.8, 26.2, 24.9, 21.4, 19.7, 12.6. FT-IR: 2945, 1716, 1694, 1443, 1282, 1227, 1156, 987, 864, 740, 662, 525 cm-1. HRMS (ESI) *m/z*: calcd for C_27_H_33_O_5_ (M+H^+^) 437.2328, found: 437.2318.

***5-(((1S,4aR,5S,8aR)-5-(methoxycarbonyl)-5,8a-dimethyl-2-methylenedecahydronaphthalen-1-yl)methyl)-6-methylnaphthalene-1,4-diyl diacetate (10)***

Na_2_S_2_O_4_ (618 mg, 3.55 mmol) was added to a suspension of quinone **7** (300 mg, 0.71 mmol) in 20 mL of H_2_O–CHCl_3_ (1:1), and the mixture was stirred for 4 h, at which time TLC showed no starting material. Then, CHCl_3_ was removed under vacuum, and the mixture was diluted with ether (30 mL), and the phases were shaken and separated. The organic layer was washed with water (10 mL) and brine (10 mL) and dried over Na_2_SO_4_. Removal of the solvent under vacuum afforded a crude product, which was used in the next step without purification. Then, the mixture resulting from the reduction of quinone **7** in pyridine (2 mL) was added at 0 °C acetic anhydride (1 mL) and DMAP (10 mg, 0.08 mmol), and the reaction mixture was stirred at room temperature for 1 h, at which time TLC showed no starting material. Then, water (2 mL) was added at 0 °C to quench the reaction, and the reaction mixture was stirred for an additional 10 min. Then, ether (50 mL) was added, and the phases were shaken and separated. The organic phase was washed with 2 N HCl solution (5 × 10 mL), water (20 mL), saturated aq NaHCO_3_ (5 × 10 mL), and brine and dried over Na_2_SO_4_. Removal of the solvent under vacuum afforded a crude product which was purified by flash chromatography on silica gel (eluting with Hexane/ethyl acetate = 8/2) to yield 299 mg of **10** (96%) as a white solid.

[**α]_D_^25^=** -13.3 (c 0.2 g/100 mL, CHCl_3_). ^1^H NMR (400 MHz, CDCl_3_) δ 7.50 (d, *J* = 8.6 Hz, 1H), 7.09 (d, *J* = 8.7 Hz, 1H), 7.02 (d, *J* = 8.2 Hz, 1H), 6.92 (d, *J* = 8.2 Hz, 1H), 4.69 (s, 1H), 4.54 (s, 1H), 3.43 (s, 3H), 3.24 (dd, *J* = 15.1, 3.2 Hz, 1H), 3.16 (dd, *J* = 15.1, 8.9 Hz, 1H), 2.36 (s, 3H), 2.28 (s, 3H), 2.23 (m, 1H), 2.16 (s, 3H), 1.87 (dd, *J* = 13.0, 4.4 Hz, 1H), 1.83 – 1.72 (m, 2H), 1.61 (qd, *J* = 13.1, 4.1 Hz, 1H), 1.51 – 1.35 (m, 2H), 1.02(m, 1H), 0.92 (s, 3H), 0.90 – 0.72 (m, 3H), 0.62 (td, *J* = 13.7, 4.2 Hz, 1H), 0.53 (s, 3H). ^13^C NMR (101 MHz, CDCl_3_) δ = 177.7, 170.0, 169.2, 152.1, 144.7, 144.4, 136.4, 135.8, 130.4, 127.8, 127.2, 119.5, 119.2, 116.3, 106.8, 56.6, 56.2, 51.1, 44.3, 41.8, 38.9, 38.5, 37.7, 28.9, 28.7, 26.3, 22.1, 21.8, 21.1, 19.8, 12.8. FT-IR: 2942, 1763, 1719, 1370, 1183, 1132, 1011, 888, 820, 750, 668, 596 cm^-1^. HRMS (ESI) *m/z*: calcd for C_31_H_39_O_6_ (M+H^+^) 507.2747, found: 507.2751.

***(1S,4aR,5S,8aR)-methyl 5-((3-hydroxy-5,8-dioxo-5,8-dihydronaphthalen-1-yl)methyl)-1,4a-dimethyl-6-methylenedecahydronaphthalene-1-carboxylate (14)***

To a stirred solution of ketone **12** (600 mg, 1.88 mmol) in dry dichloromethane (20 ml) cooled at 0 ºC, were added successively dropwise under argon atmosphere, N,N-diisopropylethylamine (0.68 ml, 3.77 mmol) and trimethylsilyl trifluoromethanesulfonate (0.34 mL, 1.88 mmol). After 30 min, sat. aqueous NaHCO_3_ solution (10 ml) was added and the aqueous phase was extracted with dichloromethane (2 x 15 ml) and the combined organic phases were dried over Na_2_SO_4_. Removal of the solvent under reduced pressure gave diene **13**, which was used in the next step without purification.

To a solution of diene **13** (734 mg, 1.88 mmol) in toluene (5 mL) was added 1,4-benzoquinone (410 mg, 3.76 mmol) and the mixture was stirred at reflux for 10 h (monitored by TLC). Then, toluene was removed under vacuum and 2,3-dichloro-5,6-dicyano-1,4-benzoquinone (934 mg, 4.14 mmol) and dioxane (10 ml) were added and the mixture was stirred at reflux for 6 h. The solvent was removed under vacuum to give a crude product which was purified by column chromatography (hexane: ethyl acetate, 8:2), obtaining compound **14** as a yellow solid (723 mg, 91%).

[**α]_D_^25^=** +38.6 (c 0.2 g/100 mL, CHCl_3_). ^1^H NMR (500 MHz, CDCl_3_) δ 7.43 (d, *J* = 2.6 Hz, 1H), 7.01 (d, *J* = 2.6 Hz, 1H), 6.87 (s, 2H), 6.18 (br s, 1H), 4.81 (d, *J* = 0.8 Hz, 1H), 4.37 (s, 1H), 3.68 (s, 3H), 3.51 (dd, *J* = 17.9, 11.0 Hz, 1H), 3.39 (dd, *J* = 17.9, 2.2 Hz, 1H), 2.45 (m, 1H), 2.33 – 2.18 (m, 2H), 2.12 – 2.02 (m, 2H), 1.99 (d, *J* = 13.6 Hz, 1H), 1.90 (ddd, *J* = 16.8, 11.5, 4.4 Hz, 2H), 1.57 (m, 1H), 1.46 (dd, *J* = 12.5, 2.6 Hz, 1H), 1.24 (s, 3H), 1.20 (dd, *J* = 13.1, 4.0 Hz, 1H), 1.10 (td, *J* = 13.4, 4.0 Hz, 1H), 0.74 (s, 3H). ^13^C NMR (126 MHz, CDCl_3_) δ = 186.1, 185.6 177.8, 159.3, 149.4, 147.6, 141.5, 135.9, 135.8, 123.5, 121.7, 111.5, 108.8, 56.6, 54.6, 51.3, 44.4, 40.8, 39.4, 38.5, 38.2, 29.0, 28.3, 26.2, 20.0, 13.1. FT-IR: 3340, 2942, 1648, 1596, 1566, 1448, 1323, 1203, 1159, 1096, 1059, 894, 842, 752 cm^-1^. HRMS (ESI) *m/z*: calcd for C_26_H_31_O_5_ (M+H^+^) 423.2171, found: 423.2167.

***(1S,4aR,5S,8aR)-methyl 5-((3-methoxy-5,8-dioxo-5,8-dihydronaphthalen-1-yl)methyl)-1,4a-dimethyl-6-methylenedecahydronaphthalene-1-carboxylate (15)***

Potassium carbonate (263 mg, 1.9 mmol) and methyl iodide (0.12 mL, 1.9 mmol) were added to a stirred solution of **14** (400 mg, 0.95 mmol) in acetone (30 mL) and the reaction mixture was stirred at reflux for 12 h, at which time TLC showed no starting material. The mixture was concentrated in vacuum to give a crude product, which was diluted with ether–water (50–20 mL). The organic phase was washed with water and brine and dried over anhydrous Na_2_SO_4_. Removal of the solvent under vacuum afforded a crude product which was purified by flash chromatography on silica gel (hexane: ethyl acetate, 8:2) affording pure **15** (829 mg, 97%) as a yellow solid.

[α] **_D_^25^**= +30.0 (c 0.6 g/100 mL, CHCl_3_). ^1^H NMR (500 MHz, CDCl_3_) δ 7.38 (d, *J* = 2.7 Hz, 1H), 6.96 (d, *J* = 2.7 Hz, 1H), 6.09 (dd, *J* = 10.2 Hz, 2H), 4.72 (s, 1H), 4.28 (s, 1H), 3.84 (s, 3H), 3.58 (s, 3H), 3.40 (m, 1H), 3.31 (dd, *J* = 17.8, 2.5 Hz, 1H), 2.35 (dt, *J* = 6.4, 4.1 Hz, 1H), 2.16 (dd, *J* = 22.2, 12.0 Hz, 2H), 2.01 – 1.87 (m, 3H), 1.86 – 1.73 (m, 2H), 1.37 (dd, *J* = 12.7, 2.9 Hz, 1H), 1.27 – 1.16 (m, 2H), 1.15 (s, 3H), 1.01 (td, *J* = 13.5, 4.0 Hz, 1H), 0.65 (s, 3H). ^13^C NMR (126 MHz, CDCl_3_) δ = 186.3, 185.6, 18.70, 162.6, 148.5, 147.6, 141.3, 136.0, 135.8, 123.5, 121.9, 108.7, 108.2, 56.6, 55.7, 54.6, 51.2, 44.4, 40.8, 39.4, 38.4, 38.3, 28.9, 28.3, 26.2, 20.0, 13.1. FT-IR: 2940, 1722, 1650, 1590, 1470, 1304, 1211, 1156, 1099, 1030, 845, 752 cm^-1^. HRMS (ESI) *m/z*: calcd for C_27_H_33_O_5_ (M+H^+^) 437.2328, found: 437.2337.

***(1S,4aR,5S,8aR)-methyl 1,4a-dimethyl-6-methylene-5-((E)-4-methylpenta-2,4-dien-1-yl)decahydronaphthalene-1-carboxylate (16)***

To a solution of MePPh_3_Br (1.35 g, 3.77 mmol) in anhydrous THF (20 mL) was added BuLi (1.5 mL, 3.77 mmol) under argon atmosphere a 0 ºC. The resulting mixture was stirred for 1 h, and then a solution of ketone **12** (1g, 3.14 mmol) was added. After 3h, the reaction was quenched with water and concentrated in vacuo. The crude product was dissolved in EtOAc and washed with water and brine. Then, the organic phase was dried over over anhydrous Na_2_SO_4_ and purified by flash chromatography on silica gel (hexane: ethyl acetate, 9:1) to afford the diene **16** as colorless oil (942 mg, 95%).

[**α]_D_^25^=** +36.3 (c 12.1 g/100 mL, CHCl_3_). ^1^H NMR (400 MHz, CDCl_3_) δ 6.04 (d, *J* = 15.8 Hz, 1H), 5.55 (dt, *J* = 15.8, 6.7 Hz, 1H), 4.79 (s, 1H), 4.75 (s, 2H), 4.49 (s, 1H), 3.54 (s, 3H), 2.32 (m, 1H), 2.25 (m, 1H), 2.16 – 2.04 (m, 2H), 1.90 (m, 1H), 1.86 (m, 1H), 1.78 (d, *J* = 13.4 Hz, 1H), 1.72 (s, 3H), 1.67 (dd, *J* = 10.3, 3.0 Hz, 1H), 1.46 (m, 1H), 1.26 (dd, *J* = 12.4, 2.9 Hz, 1H), 1.16 (m, 1H), 1.12 (s, 3H), 1.04 (dd, *J* = 17.9, 4.3 Hz, 1H), 0.96 (dd, *J* = 13.3, 4.4 Hz, 1H), 0.80 (m, 1H), 0.46 (s, 3H). ^13^C NMR (101 MHz, CDCl_3_) δ = 177.7, 147.9, 142.3, 133.2, 131.1, 113.9, 107.6, 56.3, 56.3, 51.1, 44.3, 40.2, 39.3, 38.6, 38.3, 28.9, 27.5, 26.1, 20.0, 18.7, 12.6. FT-IR: 2945, 1719, 1446, 1230, 1153, 981, 749, 664 cm^-1^. HRMS (ESI) *m/z*: calcd for C_21_H_33_O_2_ (M+H^+^) 317.2481, found: 317.2472.

***(1S,4aR,5S,8aR)-methyl 1,4a-dimethyl-5-((3-methyl-5,8-dioxo-5,8-dihydronaphthalen-1-yl)methyl)-6-methylenedecahydronaphthalene-1-carboxylate (17)***

To a solution of diene **16** (800 mg, 2.53 mmol) in toluene (8 mL) was added 1, 4-benzoquionone (546 mg, 5.06 mmol) and the mixture was stirred at reflux for 11 h (monitored by TLC). Then, solvent was removed under reduced pressure and dioxane (15 ml) and 2,3-dichloro-5,6-dicyano-1,4-benzoquinone (1.26 g, 5.57 mmol) were added. The mixture was stirred at reflux for 12 h and the solvent was removed under vacuum to give a crude product which was purified by column chromatography (hexane: ethyl acetate, 8:2), obtaining compound **17** as a yellow solid (979 mg, 92 %).

[**α]_D_^25^=** +9.1 (c 13.2 g/100 mL, CHCl_3_). ^1^H NMR (500 MHz, CDCl_3_) δ 7.77 (s, 1H), 7.34 (s, 1H), 6.86 (s, 2H), 4.77 (s, 1H), 4.36 (s, 1H), 3.65 (s, 3H), 3.49 (dd, *J* = 17.4, 10.9 Hz, 1H), 3.38 (d, *J* = 2.8 Hz, 1H), 2.42 (s, 3H), 2.29 (br d, *J* = 8.1 Hz, 1H), 2.21 (br d, *J* = 12.2 Hz, 1H), 2.10 – 1.93 (m, 3H), 1.94 – 1.82 (m, 3H), 1.56 (m, 1H), 1.47 (dd, *J* = 12.6, 2.7 Hz, 1H), 1.25 (m, 1H), 1.22 (s, 3H), 1.10 (td, *J* = 13.4, 4.0 Hz, 1H), 0.73 (s, 3H). ^13^C NMR (126 MHz, CDCl_3_) δ = 187.3, 185.8, 177.7, 147.8, 145.8, 143.5, 140.9, 136.4, 136.1, 133.5, 127.3, 125.6, 108.6, 56.6, 54.8, 51.2, 44.4, 40.8, 39.4, 38.5, 38.3, 28.9, 28.1, 26.2, 21.9, 20.1, 13.0. FT-IR: 2942, 1719, 1656, 1599, 1304, 1227, 1156, 1039, 842, 752, 667, 542, 446 cm^-1^. HRMS (ESI) *m/z*: calcd for C_27_H_33_O_4_ (M+H^+^) 421.2379, found: 421.2375.

***(1S,4aR,5S,8aR)-methyl 1,4a-dimethyl-6-methylene-5-((E)-penta-2,4-dien-1-yl)decahydronaphthalene-1-carboxylate (18)***

To a solution of diethyl allylphosphonate (1.14 g, 6.41 mmol) in THF (20 mL) at 0 ºC was added NaH (154 mg, 6.41 mmol). The mixture was stirred at 0ºC for 30 min and then, aldehyde **11** (1.5 g, 5.34 mmol) was added and the ice bath was removed. After 1h, the reaction was quenched with water (5 ml) and concentrated in vacuum. The crude product was dissolved in EtOAc (40 mL) and washed with water (15 mL) and brine (15 mL). Then, the organic phase was dried over anhydrous Na_2_SO_4_ and purified by flash chromatography on silica gel (hexane: ethyl acetate, 9:1) to afford the diene **18** (1.44 g, 90%)

[**α]_D_^25^=** +45.9 (c 5.5 g/100 mL, CHCl_3_). ^1^H NMR (500 MHz, CDCl_3_) δ 6.29 (m, 1H), 6.05 (dd, *J* = 15.3, 10.4 Hz, 1H), 5.70 (dt, *J* = 15.3, 6.8 Hz, 1H), 5.07 (d, *J* = 17.1 Hz, 1H), 4.95 (d, *J* = 11.1 Hz, 1H), 4.89 (s, 1H), 4.57 (s, 1H), 3.64 (s, 3H), 2.42 (ddd, *J* = 12.3, 4.0, 2.4 Hz, 1H), 2.34 (dd, *J* = 15.3, 7.0 Hz, 1H), 2.24 – 2.17 (m, 2H), 2.07 – 1.92 (m, 2H), 1.91 – 1.83 (m, 2H), 1.81 (dd, *J* = 12.8, 4.1 Hz, 1H), 1.75 (dd, *J* = 11.1, 2.6 Hz, 1H), 1.55 (m, 1H), 1.35 (dd, *J* = 12.6, 3.0 Hz, 1H), 1.21 (s, 3H), 1.19 – 0.98 (m, 2H), 0.55 (s, 3H). ^13^C NMR (126 MHz, CDCl_3_) δ = 177.8, 147.9, 137.4, 135.6, 131.4, 114.4, 107.6, 56.3, 56.1, 51.2, 44.3, 40.2, 39.3, 38.6, 38.3, 28.9, 27.3, 26.1, 20.0, 12.6. FT-IR: 2937, 1722, 1446, 1227, 1151, 970, 888, 752, 664 cm^-1^. HRMS (ESI) *m/z*: calcd for C_20_H_31_O_2_ (M+H^+^) 303.2324, found: 303.2315.

***(1S,4aR,5S,8aR)-methyl 5-((5,8-dioxo-5,8-dihydronaphthalen-1-yl)methyl)-1,4a-dimethyl-6-methylenedecahydronaphthalene-1-carboxylate (19)***

To a solution of diene **18** (750 mg, 2.48 mmol) in toluene (8 mL) was added 1, 4-benzoquionone (536 mg, 4.96 mmol) and the mixture was stirred at reflux for 12 h. Then, solvent was removed under reduced pressure and dioxane (15 ml) and 2,3-dichloro-5,6-dicyano-1,4-benzoquinone (1.24 g, 5.46 mmol) were added. The mixture was stirred at reflux for 12 h and the solvent was removed under vacuum to give a crude product which was purified by column chromatography (hexane: ethyl acetate, 8:2), obtaining compound **19** as a yellow solid (1.01 g, 94 %).

[**α]_D_^25^=** +30.9 (c 1.1 g/100 mL, CHCl_3_). ^1^H NMR (500 MHz, CDCl_3_) δ 7.91 (dd, *J* = 6.6, 2.3 Hz, 1H), 7.60 – 7.44 (m, 2H), 6.82 (s, 2H), 4.70 (s, 1H), 4.26 (s, 1H), 3.58 (s, 3H), 3.41 (dd, *J* = 17.7, 10.5 Hz, 1H), 3.34 (dd, *J* = 17.7, 3.1 Hz, 1H), 2.40 – 2.29 (m, 1H), 2.22 (d, *J* = 8.1 Hz, 1H), 2.14 (d, *J* = 13.5 Hz, 1H), 2.00 (m, 1H), 1.95 – 1.88 (m, 2H), 1.87 – 1.73 (m, 2H), 1.53 – 1.45 (m, 2H), 1.38 (dd, *J* = 12.5, 2.7 Hz, 1H), 1.15 (s, 3H), 1.02 (td, *J* = 13.5, 4.0 Hz, 1H), 0.66 (s, 3H). ^13^C NMR (126 MHz, CDCl_3_) δ = 187.59, 185.49, 177.70, 147.78, 145.54, 140.82, 136.58, 135.57, 133.51, 132.67, 129.57, 124.97, 108.75, 56.62, 54.66, 51.26, 44.40, 40.84, 39.40, 38.49, 38.25, 28.95, 28.28, 26.23, 20.06, 13.05. FT-IR: 2945, 1724, 1659, 1585, 1293,1156, 1093, 1036, 850, 752, 667, 562, 440 cm^-1^. HRMS (ESI) *m/z*: calcd for C_26_H_31_O_4_ (M+H^+^) 407.2222, found: 407.2220.

^1^H NMR spectrum of **9** (CDCl_3_, 400 MHz)

^13^C NMR spectrum of **9** (CDCl_3_, 400 MHz)
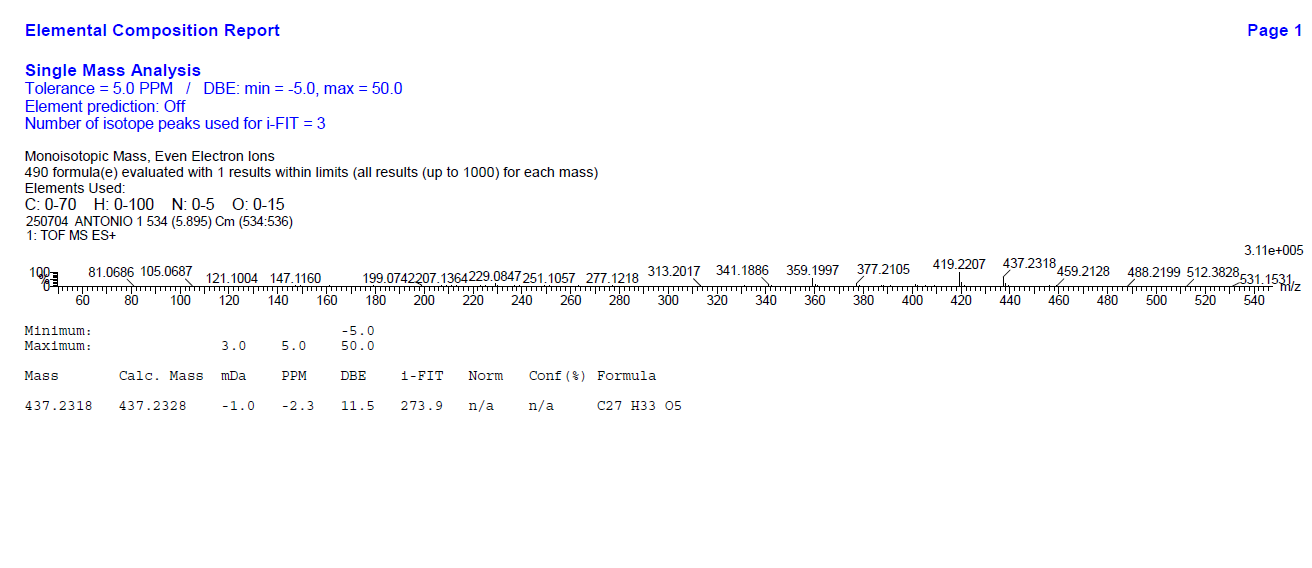


HRMS report for **9**

^1^H NMR spectrum of **10** (CDCl_3_, 400 MHz)

^13^C NMR spectrum of **10** (CDCl_3_, 400 MHz)
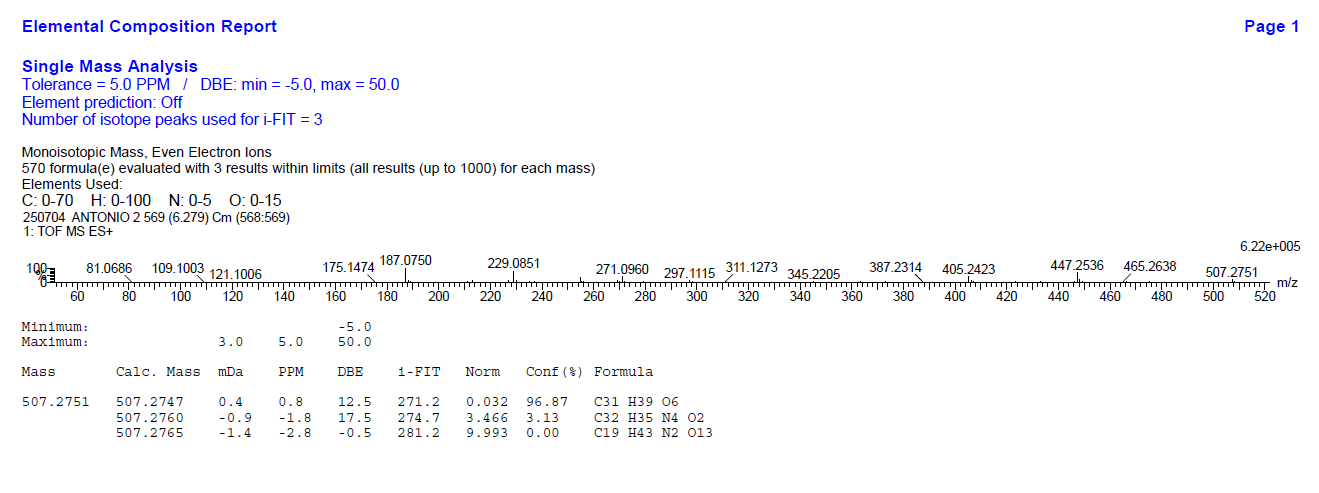

HRMS report for **10**

^1^H NMR spectrum of **14** (CDCl_3_, 400 MHz)

^13^C NMR spectrum of **14** (CDCl_3_, 400 MHz)
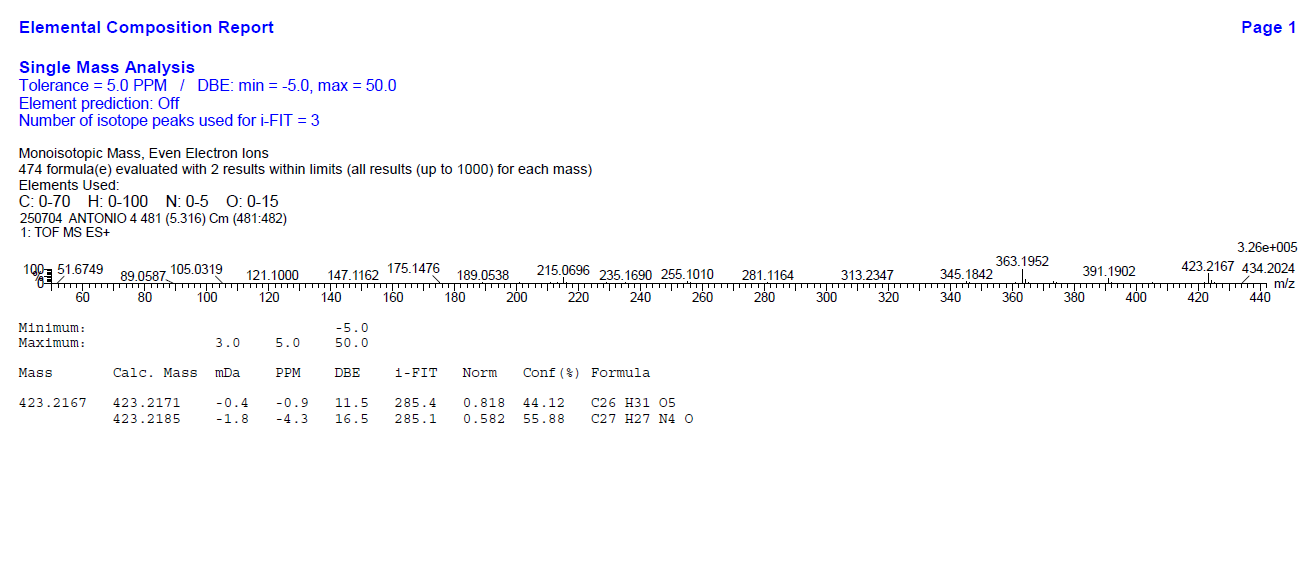


HRMS report for **14**

^1^H NMR spectrum of **15** (CDCl_3_, 400 MHz)

^13^C NMR spectrum of **15** (CDCl_3_, 400 MHz)
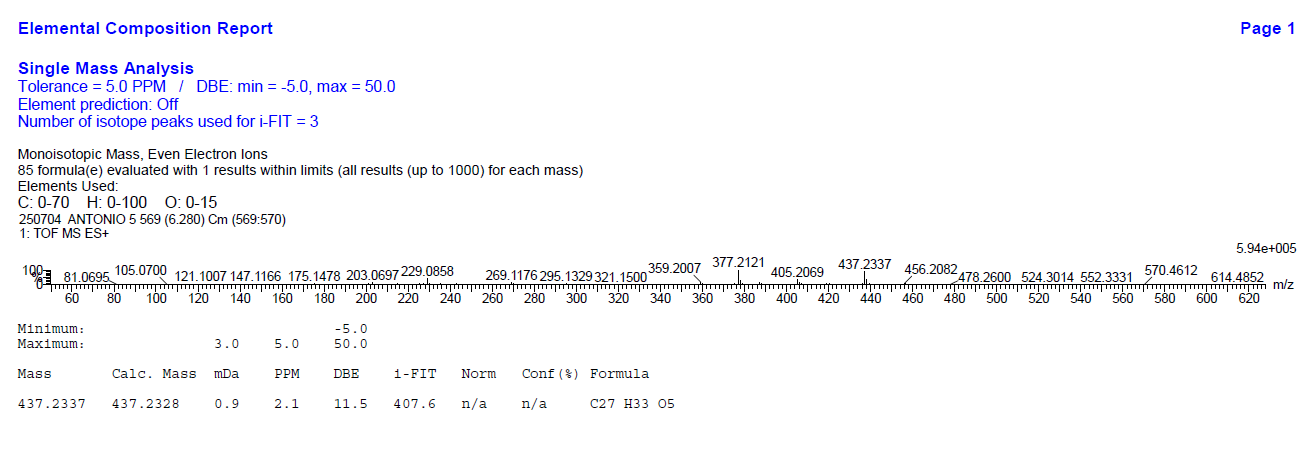

HRMS report for **15**

^1^H NMR spectrum of **16** (CDCl_3_, 400 MHz)

^13^C NMR spectrum of **16** (CDCl_3_, 400 MHz)
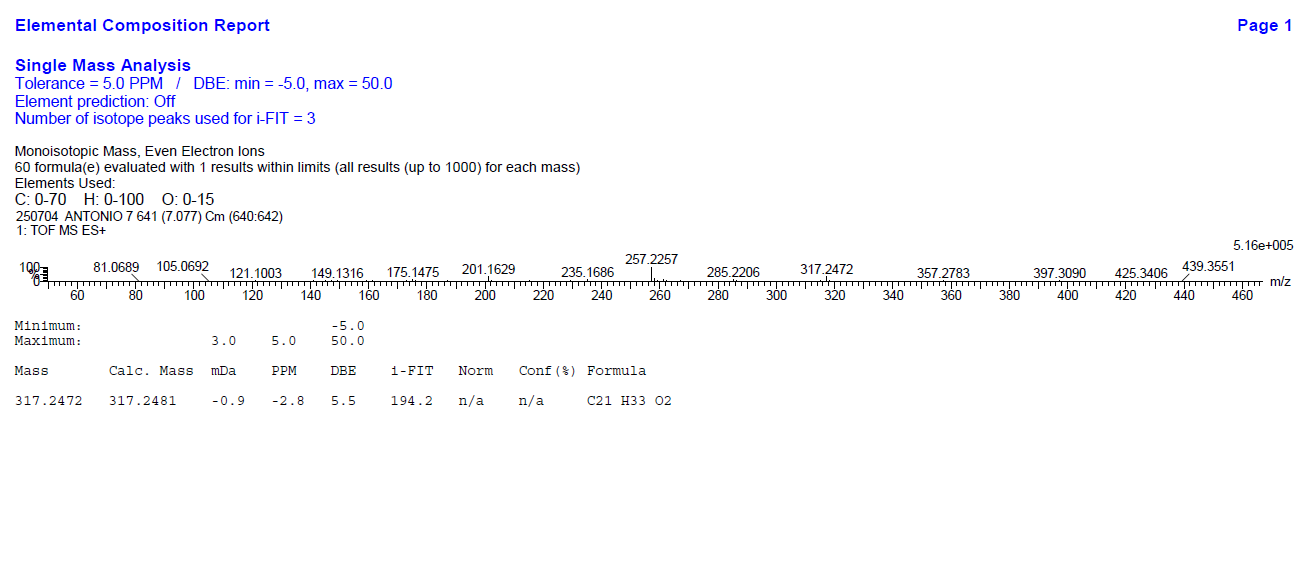


HRMS report for **16**

^1^H NMR spectrum of **17** (CDCl_3_, 400 MHz)

^13^C NMR spectrum of **17** (CDCl_3_, 400 MHz)
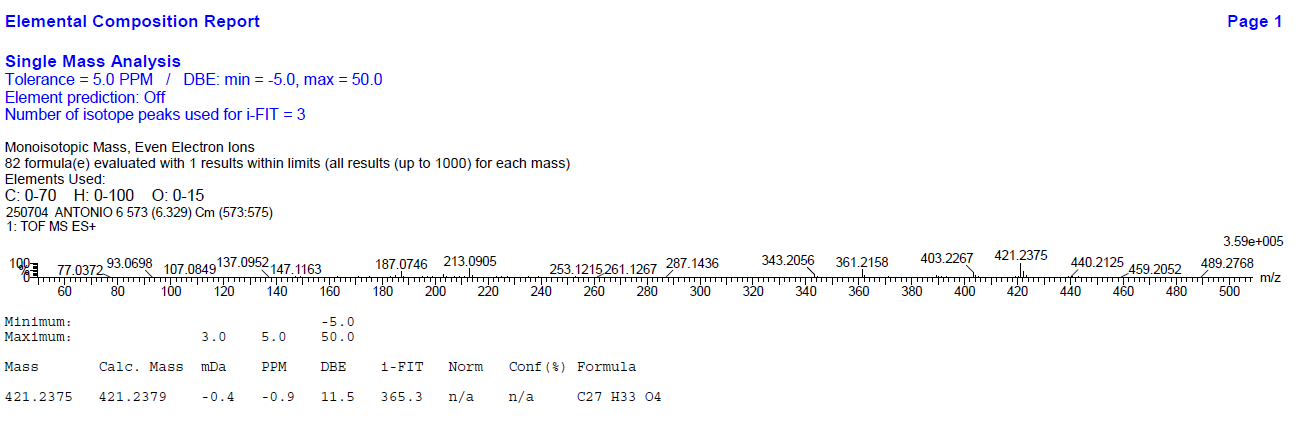

HRMS report for **17**

^1^H NMR spectrum of **18** (CDCl_3_, 400 MHz)

^13^C NMR spectrum of **18** (CDCl_3_, 400 MHz)
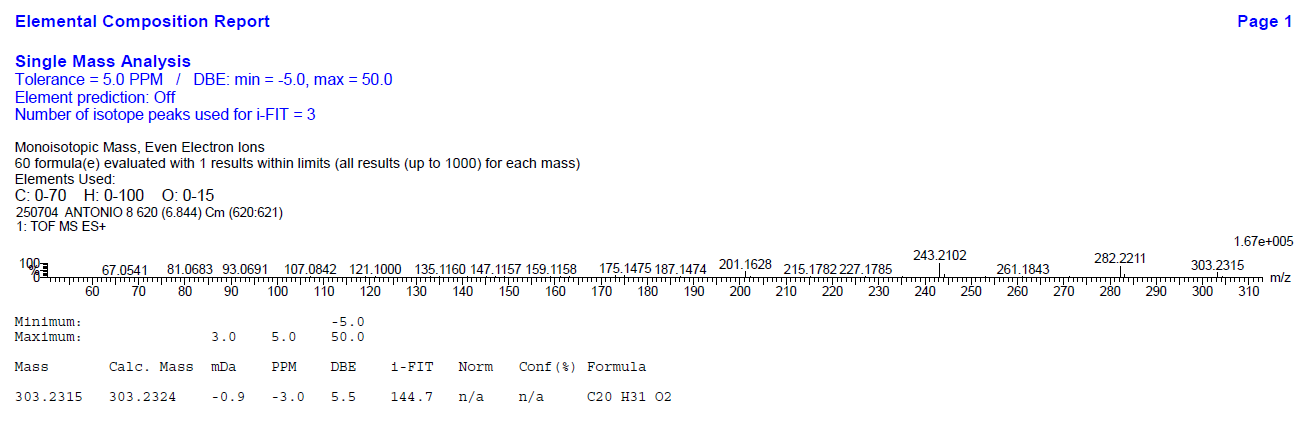

HRMS report for **18**

^1^H NMR spectrum of **19** (CDCl_3_, 400 MHz)

^13^C NMR spectrum of **19** (CDCl_3_, 400 MHz)


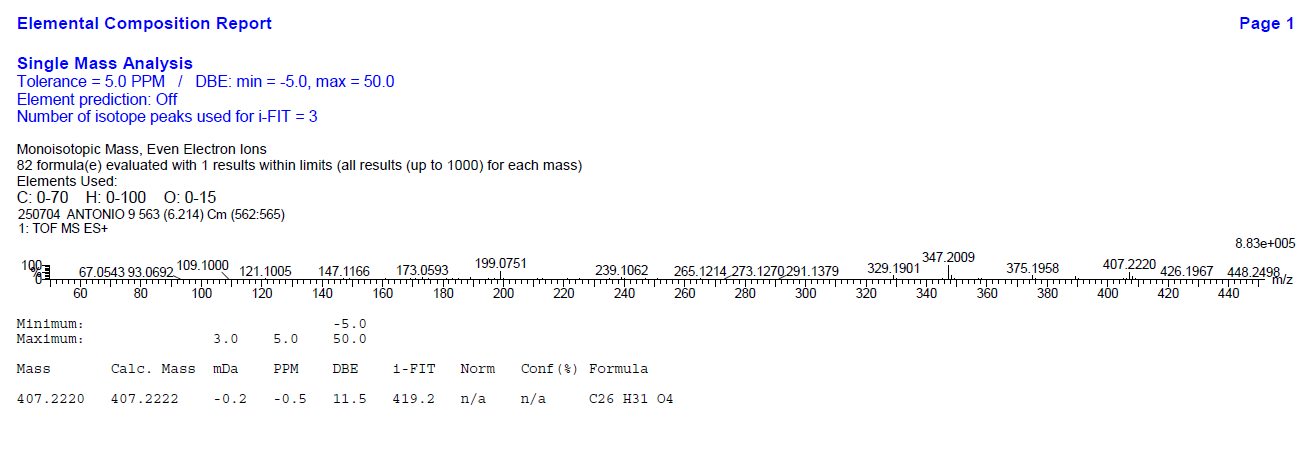

HRMS report for **19**
